# Supplementary material for: HyU: Hybrid Unmixing for longitudinal in vivo imaging of low signal-to-noise fluorescence
Source: Nat Methods. 2023 Jan 19;20(2):248–58. doi: 10.1038/s41592-022-01751-5 (PMC9911352; doi:10.1038/s41592-022-01751-5)
Supplement: Supplementary file 1 — Supplementary Methods, Supplementary Figs. 1–30, Supplementary Discussion, Supplementary Table 1 and Supplementary Notes 1–4. [file 41592_2022_1751_MOESM1_ESM.pdf]

nature methods

Article https://doi.org/10.1038/s41592-022-01751-5

HyU: Hybrid Unmixing for longitudinal in vivo imaging of low signal-to-noise fluorescence

In the format provided by the authors and unedited

Table of content

Supplementary Methods:

- **Performance quantification**
- **Residuals**

Figures:

- **S1: HyU unmixing reduces noise and signal bleedthrough compared to traditional bandpass filter imaging.**
- **S2: HyU algorithm outperforms current methods.**
- **S3: Comparison of unmixing results for synthetic data at different SNR demonstrate improved HyU performance.**
- **S4: Quantification of HyU vs LU unmixing results for synthetic data highlight increased HyU performance**
- **S5: Residual analysis for synthetic data identifies locations with reduced algorithm performance**
- **S6: Schematic overview of residual calculation.**
- **S7: Unmixing of a quadra-transgenic zebrafish with HyU and LU highlights improvements in contrast and spatial features.**
- **S8: Residual analysis of experimental data supports performance improvement of HyU.**
- **S9: Application of denoising filters reveals improved results with lower residuals.**
- **S10: Comparison of residual images for LU and HyU highlights improved HyU performance.**
- **S11: Residual maps facilitate identification of independent spectral components.**
- **S12: HyU analysis of 36 hpf Casper zebrafish demonstrates feasibility of unmixing only intrinsic signals**
- **S13: Speed comparison and improvement plots of multiple unmixing algorithms in their original form vs HyU encoded.**
- **S14: Residuals in synthetic data and experimental data.**
- **S15. HyU unmixing on low concentration signals using customized independent spectra**
- **S16. RMSE improvement for simulated fluorescent spectral combinations highlights increased HyU performance across multiple denoising filters.**

- **S17. RMSE improvement for simulated fluorescent and autofluorescent spectral combinations highlights increased HyU performance across multiple denoising filters**
- **S18. RMSE improvement for simulated fluorescent spectral combinations highlights decreasing overall performance across decreased number of spectral channels**
- **S19. RMSE improvement for simulated fluorescent and autofluorescent spectral combinations highlights decreasing overall performance across decreased number of spectral channels**
- **S20. Emission spectra of components in overlapping simulation**
- **S21. Pre-identified positions for common fluorophores on the phasor map**
- **S22. FAD autofluorescence in high magnification hindbrain region of zebrafish embryo**
- **S23. Phasor analysis on signal distortion in deep tissue**
- **S24. Comparison of HyU vs HySP results from a spectrally overlapping and spatially disperse sample**
- **S25. Comparison of HyU vs HySP results from a spectrally overlapping and spatially non-disperse sample**
- **S26. Residual analysis of intrinsic fluorescent signals of HyU and LU shows robust results for HyU unmixing**
- **S27. Endmember Spectrum selection process**
- **S28. Relationship between Spectral SNR and Photon/Spectrum**
- **S29 Intrinsic fluorescent signatures in fresh mouse tissue**
- **S30 Extrinsic fluorescent signatures in fixed mouse tissue**

Tables:

- **S1 data**

Movies:

- **V1: HyU vs LU unmixing results**
- **V2: 3 colors 300 mins time-lapse video**
- **V3: 9 colors time-lapse video with intrinsic and extrinsic signals**

Notes:

- **N1: Identification of spectra and new components with HyU**
- **N2: HyU in autofluorescent data**
- **N3: Reduced computational costs during unmixing**
- **N4: Improvements of HyU over the standard phasor analysis**

Supplementary Methods:

Performance quantification

Mean Square Error

For synthetic data, a ground truth is available for comparison of unmixing fidelity between HyU and LU. *fp* contributions, or ratios, were used for quantification, owing to the arbitrary nature of intensity values in microscopy data. We utilize Mean Square Error (MSE) for determining the quality of the ratios in synthetic data. We define *MSE* as the square difference of the ratio recovered by an unmixing algorithm ($r_{unmixed}$) and the ground truth ratio (r) divided by the total number of pixels (n).

$$MSE = \frac{1}{n} |r_{unmixed} - r|^2 \quad (1)$$

To simplify comparison between different unmixing algorithms, we define Relative Mean Square Error (*RMSE*) as:

$$RMSE = \left(\frac{MSE_{LU}}{MSE_{HyU}} - 1\right) * 100\% \quad (2)$$

RMSE measures the improvement in *MSE* when using HyU as compared to LU.

Residuals

For experimental data, in the absence of ground truth, we quantify the performance of the results returned by the unmixing algorithms with the following measurements: Average Relative Residual, Residual Image Map, Residual Phasor Map, and finally, Residual Intensity Histogram.

Residual (R) is calculated as:

For image: $R(x, y, c, z, t) = I_{Raw\ Image}(x, y, c, z, t) - I_{Unmixed\ Image}\ (x, y, c, z, t)$ (3)

For phasor: $R(g, s, c) = I_{Raw\ Image}(g, s, c) - I_{Unmixed\ Image}\ (g, s, c)$ (4)

The spectral intensity difference between the unmixed image and original image for each pixel or phasor bin depend on the following descriptions of the intensity image (I), where:

$$I_{Raw\ Image} = \sum_i r * fp_i + N \quad (5)$$

$$I_{Unmixed\ Image} = \sum_i r_{unmixed} * fp_i \quad (6)$$

The original spectrum ($I_{Raw\ Image}$) is the combination of each independent spectral component (fp) with its ratio (r) plus noise (N). The recovered spectrum is obtained by the multiplication of recovered ratios ($r_{unmixed}$) with each corresponding individual component.

Relative Residual (*RR*) is calculated as the sum of the residual values over C channels and normalized to the sum of the original intensity values over C channels (with C = 32 in our instrument).

$$RR(x, y, z, t) = \frac{\sum_{c=1}^{C} R(x,y,c,z,t)}{\sum_{c=1}^{C} I_{Raw\ Image}(x,y,c,z,t)} \quad (7)$$

$$RR(g, s) = \frac{\sum_{c=1}^{C} R(g,s,c)}{\sum_{c=1}^{C} I_{Raw\ Image}(g.s.c)} \quad (8)$$

The Average Relative Residual (Supplementary Figure 5) provides a single comparison value for evaluating the performance of different processing methods on the same data, such as the application of multiple filters, applying of various threshold values, and variations in the number of components estimated. Average Relative Residual (RR_{avg}) is defined as the average of the relative residual for every pixel in the image or every phasor bin in the phasor histogram.

$$\text{For image: } RR_{avg} = \frac{\sum_t \sum_z \sum_y \sum_x RR}{xyzt} \quad (9)$$

$$\text{For Phasor: } RR_{avg} = \frac{\sum_s \sum_g RR}{gs} \quad (10)$$

The Residual Image Map visualizes the residual values for each pixel of the image (Supplementary Figure 4). Regions with higher residual values appear to characterize portions of the dataset with increased amount of noise or where an unexpected spectral signature is present.

Residual Image Maps ($R_{img\ map}(x, y)$) project the Relative Residual (RR) cube to the 2D image shape for each voxel, providing an estimated visualization of an algorithm ratio recovery performance in the spatial context of the original image.

$$R_{img\ map}(x, y) = \sum_{z,t} RR(x, y, z, t) * 100 \quad (11)$$

Residual Phasor Map visualizes residuals for each bin of the phasor histogram (Supplementary Figure 4). These maps allow for insights on where HyU unmixing results have reduced performance in phasor domain and indicate phasor locations of unexpected additional spectral components (Supplementary Figure 5).

$$R_{ph\ map}(g, s) = RR(g, s) * 100 \quad (12)$$

The Residual Intensity Histogram $R_{Int\ Hist}(p, rr)$ (Figure 3 g,h, Supplementary Figure 4d) calculates the distribution of the relative residual in relation to intensity overall all pixels or all phasor bins. Higher residuals appear to be present in regions with lower signal intensity and SNR, providing degraded performance.

For image:

$$R_{Int\ Hist}(p, rr) = count\big(P(x, y, z, t), RR(x, y, z, t)\big)_{p,rr} \quad (13)$$

For phasor:

$$R_{Int\ Hist}(p, rr) = count\big(P(g, s), RR(g, s)\big)_{p,rr} \quad (14)$$

$$P = \frac{\sum_{c=1}^{C} I_{Raw\ Image}}{4 * sf} \quad (15)$$

Where *p* is a bin of the histogram *P*, *rr* is a bin of *RR*, and *sf* is the factor which converts the number of photons to digital intensity levels.

Image Contrast

Image contrast measures the distinguishability of a detail against the background. Here we use percent contrast to refer the relationship between the highest and lowest intensity in the image.

$$Contrast = \frac{I_S - I_B}{I_B} \quad (16)$$

Where the Intensity of signal average (I_s) is the average of top 20% intensities in the image. The Intensity of background average (I_B), the average of bottom 20% image intensities.

Spectral Signal to Noise Ratio

Since each synthetic dataset has a ground truth, the SNR can be calculated by comparing the simulated image to the ground truth. Since these are hyperspectral images, we extend the definition of SNR to the wavelength dimension of the data and use the term Spectral SNR. We define two types of Spectral SNR, Absolute Spectral SNR and Relative Spectral SNR. Spectral SNR is calculated as follows for each single spectrum simulation. First, for each pixel and channel, the absolute value of the difference is taken between the ground truth intensity and the simulated intensity. Then the mean is calculated over all of the pixels for each channel. Finally, the sum is taken over all of the channels and divided by either 32 for the absolute SNR, or the number of channels with signal for the relative SNR. The number of channels with signal is calculated by checking if there is a statistically significative number of pixels in a single channel with a pixel SNR value greater than zero.

$$Absolute\ SNR = \frac{\sum_{c=1}^{32} \frac{\sum_{n=1}^{P} \frac{i_{gnd}}{i_{sim} - i_{gnd}}}{P}}{32} \quad (17)$$

$$Relative\ SNR = \frac{\sum_{c=1}^{C} \frac{\sum_{n=1}^{P} \frac{i_{gnd}}{i_{sim} - i_{gnd}}}{P}}{C} \quad (18)$$

Where i_{gnd} is the intensity per pixel per channel for the ground truth data, i_{sim} is the intensity per pixel per channel for the simulated (noisy) data, P is the total number of pixels, and C is the number of channels with signal.

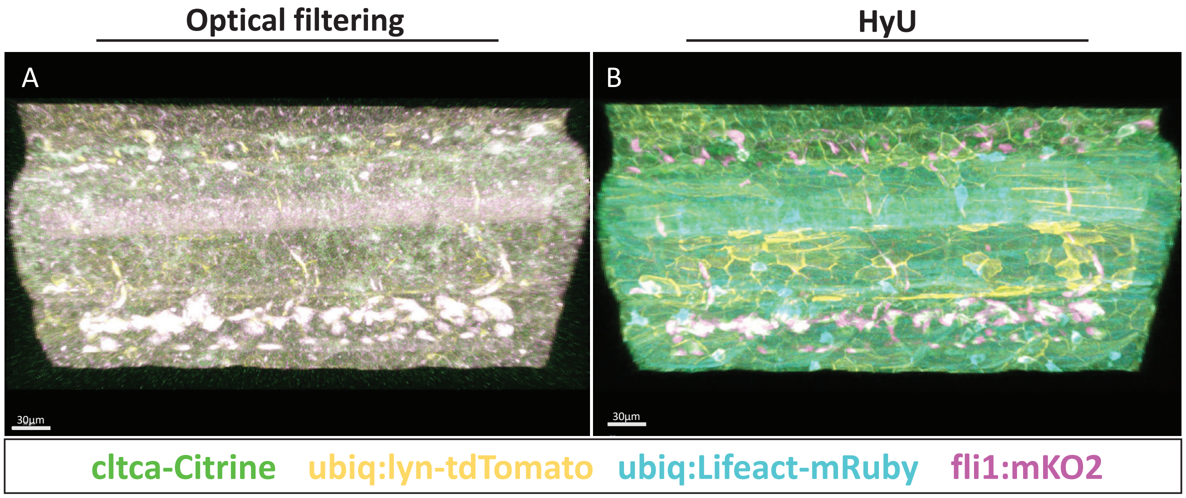
Optical filtering
HyU
A
B
30µm
30µm
cltca-Citrine
ubiq:lyn-tdTomato
ubiq:Lifeact-mRuby
fli1:mKO2

Supplementary Figure 1. HyU unmixing reduces noise and signal bleedthrough compared to traditional bandpass filter imaging. Imaging of a quadra-transgene zebrafish *Gt(cltca-citrine);Tg(ubiq:lyn-tdTomato;ubiq:Lifeact-mRuby;fli1:mKO2)* (same data as fig. 3) performed with **(A)** 4-channel optical filter imaging and **(B)** multispectral imaging and Hybrid Unmixing (HyU) analysis. Bleed-through from the fluorophores' overlapping emission spectra is present in **A**. This artifact is the result of the sharp spectral discretization imposed on the fluorescent signals by the optical filters, which fail to produce a clean distinction between Citrine (480nm-690nm), mKO2 (525nm-690nm), tdTomato (530nm-690nm) and mRuby (560nm-690nm). Fluorophores are well separated in **B**. Colors in both **A** and **B** represent Citrine (green), mKO2 (yellow), tdTomato (magenta) and mRuby (cyan)

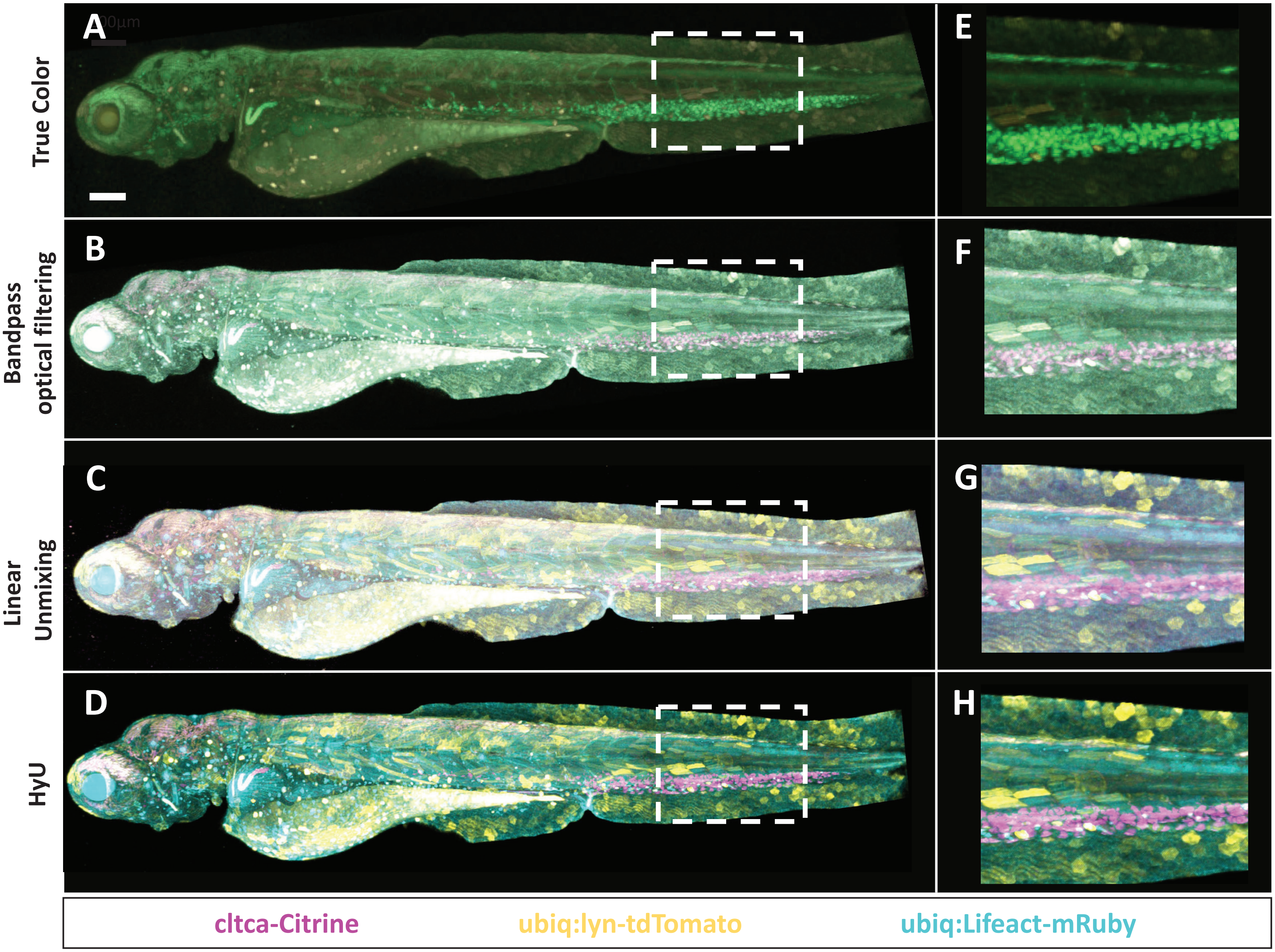

A
E
True Color
B
F
Bandpass
optical filtering
C
G
Linear
Unmixing
D
H
HyU
cltca-Citrine
ubiq:lyn-tdTomato
ubiq:Lifeact-mRuby

Supplementary Figure 2. **HyU algorithm outperforms current methods. (A)** True-color rendering of a 32 channel tetra-label *Gt(cltca-citrine);Tg(ubiq:lyn-tdTomato;ubiq:Lifeact-mRuby)* zebrafish shows indistinguishability of multi-label in the absence of analysis. **(B)** Optical filter imaging presents strong bleedthrough across the 4 channels. **(C)** Traditional unmixing provides contrast increase across labels while still affected by incorrect re-assignment of signals. **(D)** Hybrid Unmixing enhances separation of spectral and spatial overlapping signals. **(E-H)** are the zoom in from white boxes in **A**, **B**, **C**, **D** respectively. Scale bar is 100 μm.

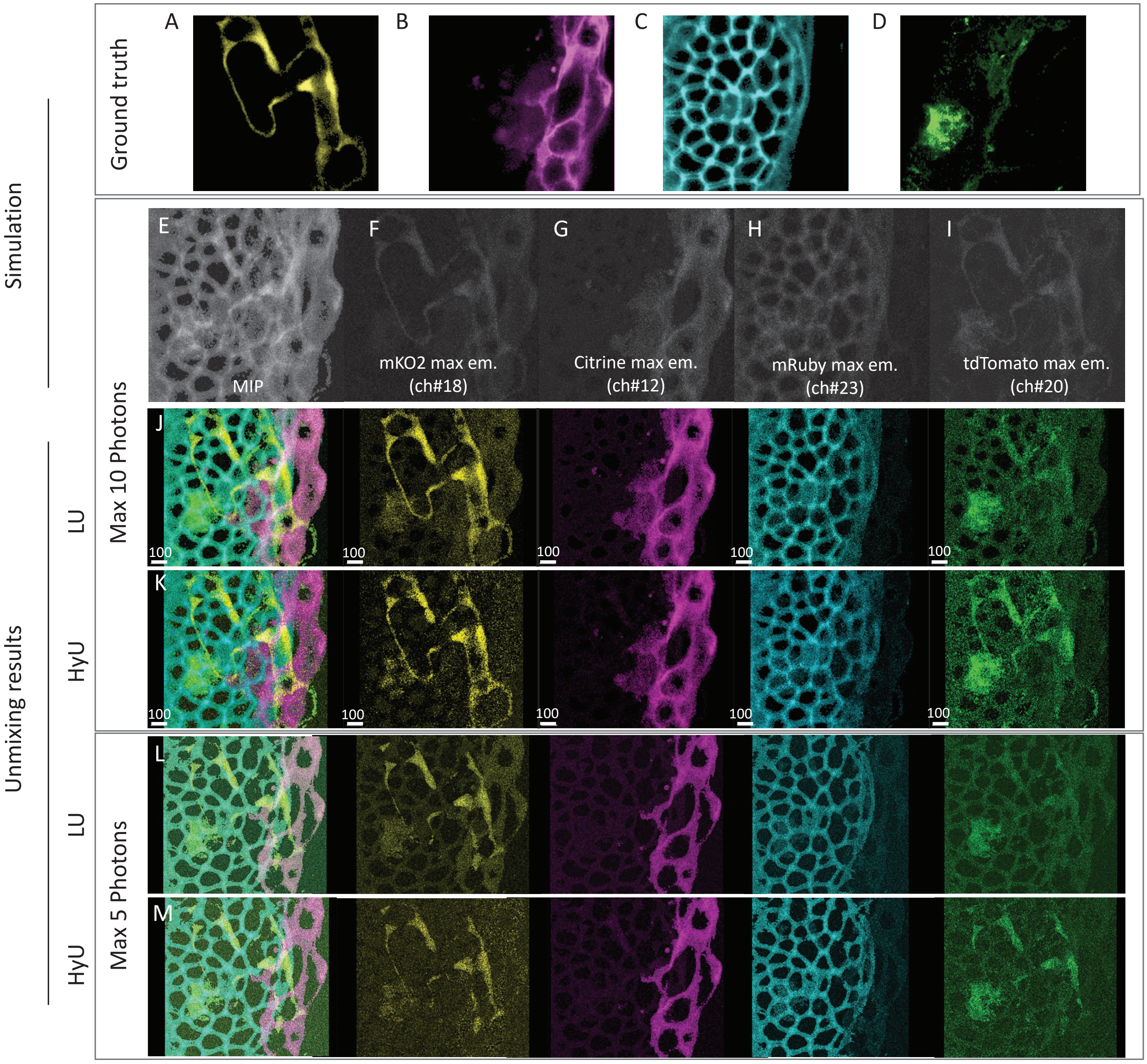
Simulation
Unmixing results
Ground truth
A
B
C
D
Max 10 Photons
Max 5 Photons
LU
HyU
E
F
G
H
I
MIP
mKO2 max em.
(ch#18)
Citrine max em.
(ch#12)
mRuby max em.
(ch#23)
tdTomato max em.
(ch#20)
J
K
L
M
100

Supplementary Figure 3. Comparison of unmixing results for synthetic data at different SNR demonstrate improved HyU performance. Ground truth photon mask of the four independent fluorescent signals, **(A)** mKO2, **(B)** Citrine, **(C)** mRuby, and **(D)** tdTomato for synthetic data. **(E)** The maximum intensity projection (MIP) of the simulated 32 channel hyperspectral image generated from the four ground truth masks at low signal-to-noise ratio (SNR). In this case, a maximum of 10 photons are simulated for each fluorescent component. (**F**-**I**) Grayscale representation of the maximum emission channel of each component, based on the respective spectra. Unmixing result of **(J)** LU and **(K)** HyU for simulations with a maximum of 10, report decreased performance. In the ultra-low SNR simulation (5 photon at most for each component), both LU **(I)** and HyU **(M)** results are deteriorated, however HyU maintains a 1.5x lower average MSE compared to LU.

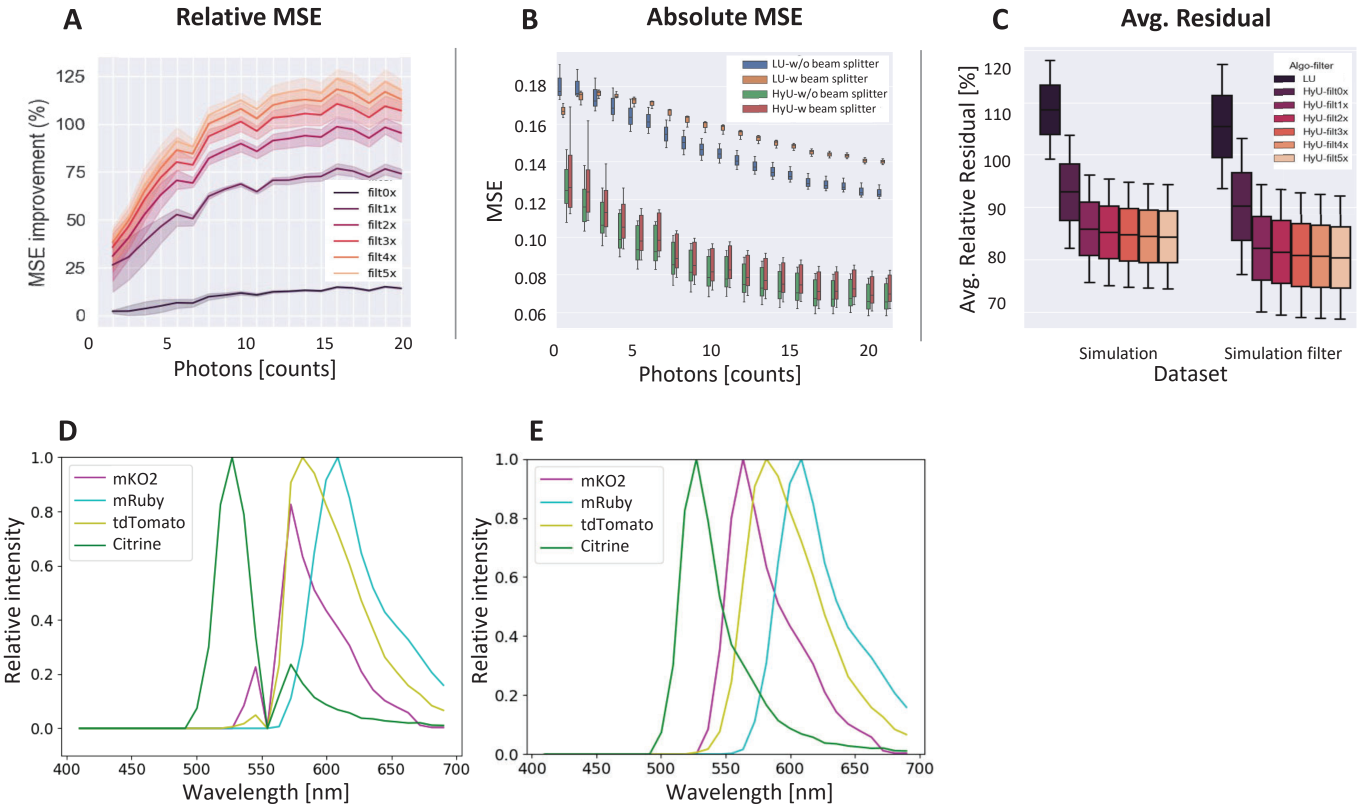
A
Relative MSE
MSE improvement (%)
Photons [counts]
filt0x
filt1x
filt2x
filt3x
filt4x
filt5x
B
Absolute MSE
MSE
Photons [counts]
LU-w/o beam splitter
LU-w beam splitter
HyU-w/o beam splitter
HyU-w beam splitter
C
Avg. Residual
Avg. Relative Residual [%]
Algo-filter
LU
HyU-filt0x
HyU-filt1x
HyU-filt2x
HyU-filt3x
HyU-filt4x
HyU-filt5x
Simulation
Simulation filter
Dataset
D
Relative intensity
Wavelength [nm]
mKO2
mRuby
tdTomato
Citrine
E
Relative intensity
Wavelength [nm]
mKO2
mRuby
tdTomato
Citrine

Supplementary Figure 4. Quantification of HyU vs LU unmixing results for synthetic data highlight increased HyU performance. HyU performance is evaluated under several algorithmic parameters and experimental conditions. **(A)** Relative MSE between HyU and LU was calculated as a function of max input photons/spectrum over 5 denoising filters for HyU. The improvement increases both with the number of photons and the number of denoising filters, showing significant differences above 7 photons/spectra with peak at 124%. Shaded regions denote the 95% confidence interval around the mean. (**B**) Absolute MSE from LU and HyU algorithms for the same synthetic dataset with and without beam splitters. The addition of optical filters causes the MSE of LU to increase on average by 8% , compared to an average increase of 5% for HyU. N = 1.05e6 pixels. Center: Median; Box: First/Third Quartile; Whiskers: 1.5x first, third quartiles; Min/max not shown. (**C**) Average relative residual of synthetic data with and without beam splitters with increasing level of denoising. The average relative residual without beam splitters with denoising (HyU-filt1x – HyU-filt5x) is 83%, compared to 109% for LU. In the absence of denoising filters (filt0x) the average relative residual is 92.9%. Beam splitters were applied in this simulation and both Mean Squared Error (MSE) and residual values were calculated with and without beam splitters. N = 1.05e6 pixels. Center: Median; Box: First/Third Quartile; Whiskers: 1.5x first, third quartiles; Min/max not shown. **(D)** Simulated spectral with beam splitter and **(E)** simulated spectral without beam splitter are shown. All box plot elements are defined as described in Methods.

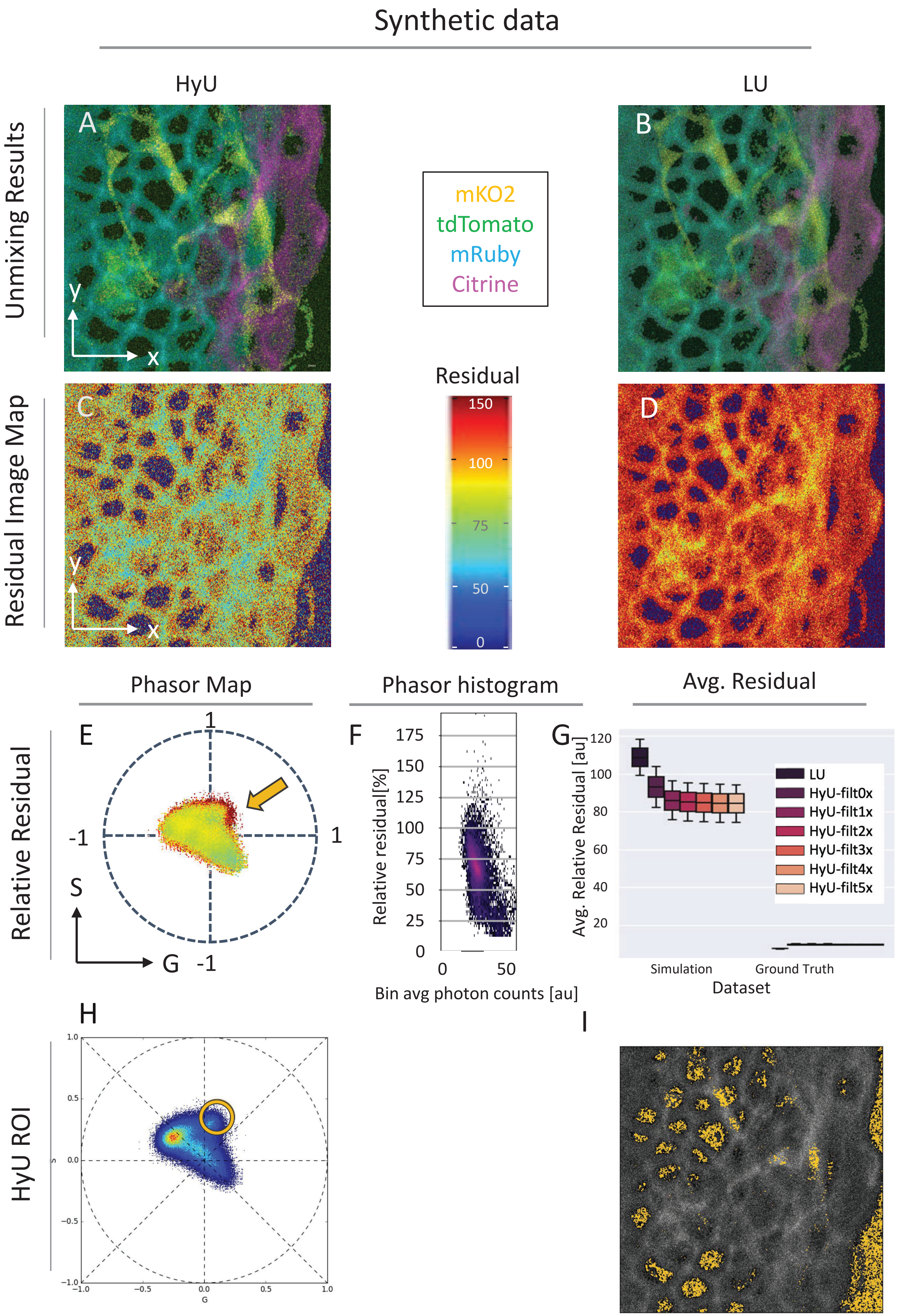

Synthetic data
HyU
LU
Unmixing Results
A
B
mKO2
tdTomato
mRuby
Citrine
Residual Image Map
C
D
Residual
150
100
75
50
0
Phasor Map
Phasor histogram
Avg. Residual
Relative Residual
E
1
-1
1
S
G
-1
F
175
150
125
100
75
50
25
0
Relative residual[%]
0
50
Bin avg photon counts [au]
G
120
100
80
60
40
20
Avg. Relative Residual [au]
LU
HyU-filt0x
HyU-filt1x
HyU-filt2x
HyU-filt3x
HyU-filt4x
HyU-filt5x
Simulation
Ground Truth
Dataset
H
HyU ROI
1.0
0.5
0.0
−0.5
−1.0
−1.0
−0.5
0.0
0.5
1.0
G
I

Supplementary Figure 5. Residual analysis for synthetic data identifies locations with reduced algorithm performance. Simulated data in Figure 2 for four fluorescent labels (Citrine, mKO2, tdTomato, mRuby) is analyzed with LU and HyU. Unmixing results for **(A)** HyU and **(B)LU**. Residual Image Map for (**C**) HyU and (**D**) LU results presents regions with higher residual (red) along the boundary between the sample's labelled features and the background, where signal-to-noise drops. The average residual values for LU (118%) are higher than HyU (94%). (**E**) Residual Phasor Map shows higher residual for the background region (arrow), consistently with the results in **C**, **D**. The Jet colorbar scale corresponds to **C**, **D** and **E**. (**F**) Phasor Residual Intensity Histogram maps the average photon counts in each histogram bin in **E** between 0 and 50 photons and presents a trend of decreasing relative residuals with photon number. The (**G**) Average Relative Residual plot shows higher values for LU compared to HyU with different denoising filters applied. Ground truth values are also included for comparison. N = 1.05e6 pixels. Center: Median; Box: First/Third Quartile; Whiskers: 1.5x first, third quartiles; Min/max not shown. An **(H)** original phasor plot with 0 threshold and 5 denoising filters applied is presented. The ROI (yellow circle) highlights the background pixels in yellow in the **(I)** average spectral intensity image. The noise from background and residual can be decreased considerably with an intensity threshold.

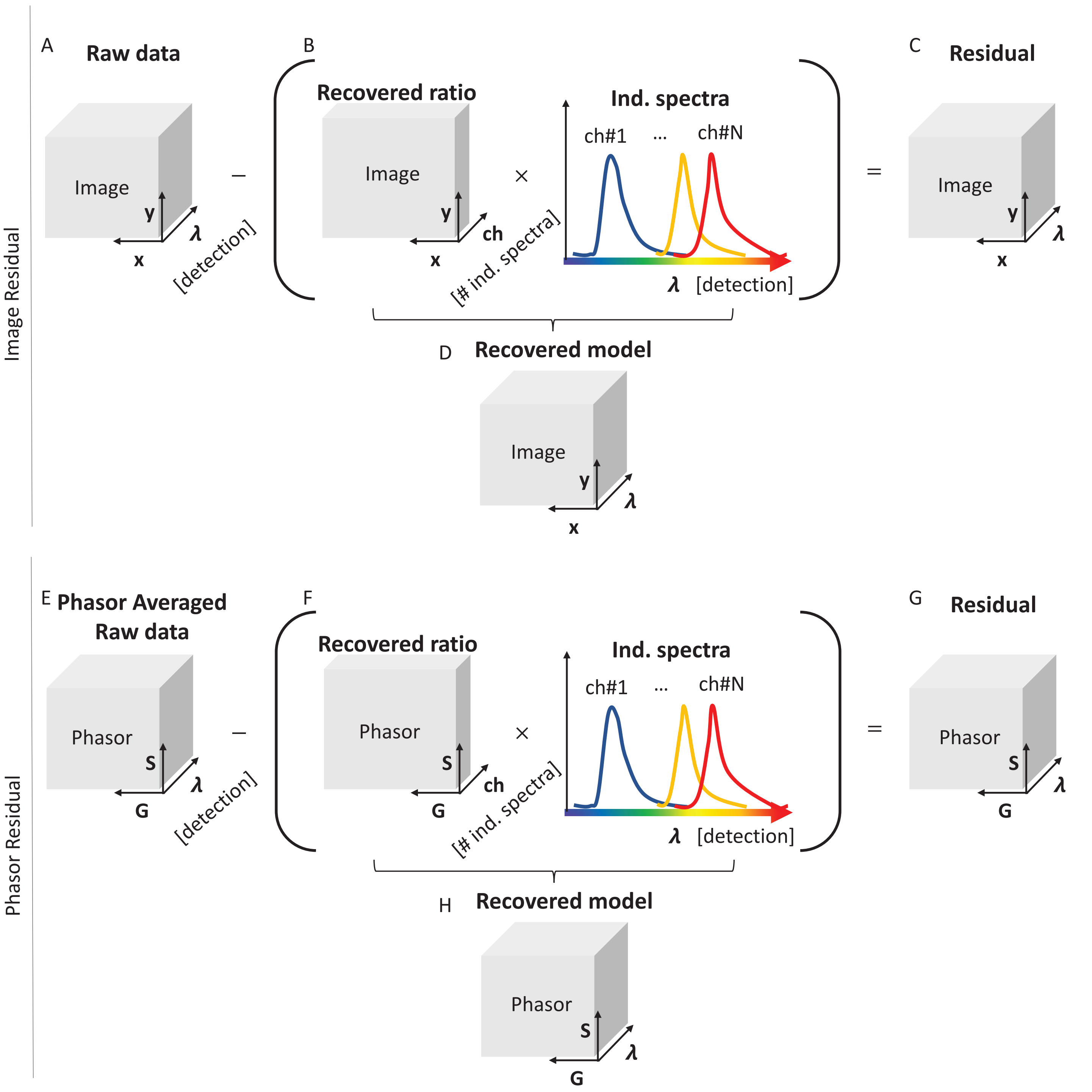
Image Residual
A
Raw data
Image
y
x
λ
[detection]
−
B
Recovered ratio
Image
y
x
ch
[# ind. spectra]
×
Ind. spectra
ch#1
...
ch#N
λ [detection]
=
C
Residual
Image
y
x
λ
D
Recovered model
Image
y
x
λ
Phasor Residual
E
Phasor Averaged
Raw data
Phasor
S
G
λ
[detection]
−
F
Recovered ratio
Phasor
S
G
ch
[# ind. spectra]
×
Ind. spectra
ch#1
...
ch#N
λ [detection]
=
G
Residual
Phasor
S
G
λ
H
Recovered model
Phasor
S
G
λ

Supplementary Figure 6. **Schematic overview of residual calculation.** Image residual is the residual for image (x, y). **(A)** Raw hyperspectral data cube in dimension of (x, y, λ). x, y is the spatial dimension. λ is the wavelength range from the spectral channel on the detector. **(D)** Recovered model in dimension of (x, y, λ) comes from **(B)** the product of Recover ratio (x, y, ch) and Independent spectra (ch, λ). ch is the number of independent spectra or unmixing component. **(C)** Residual is the difference of Recovered model and Raw data. **(E - H)** Same logic for Phasor residual, but instead of (x, y) the dimension of Phasor is composed from the real and imaginary Fourier components (G, S).

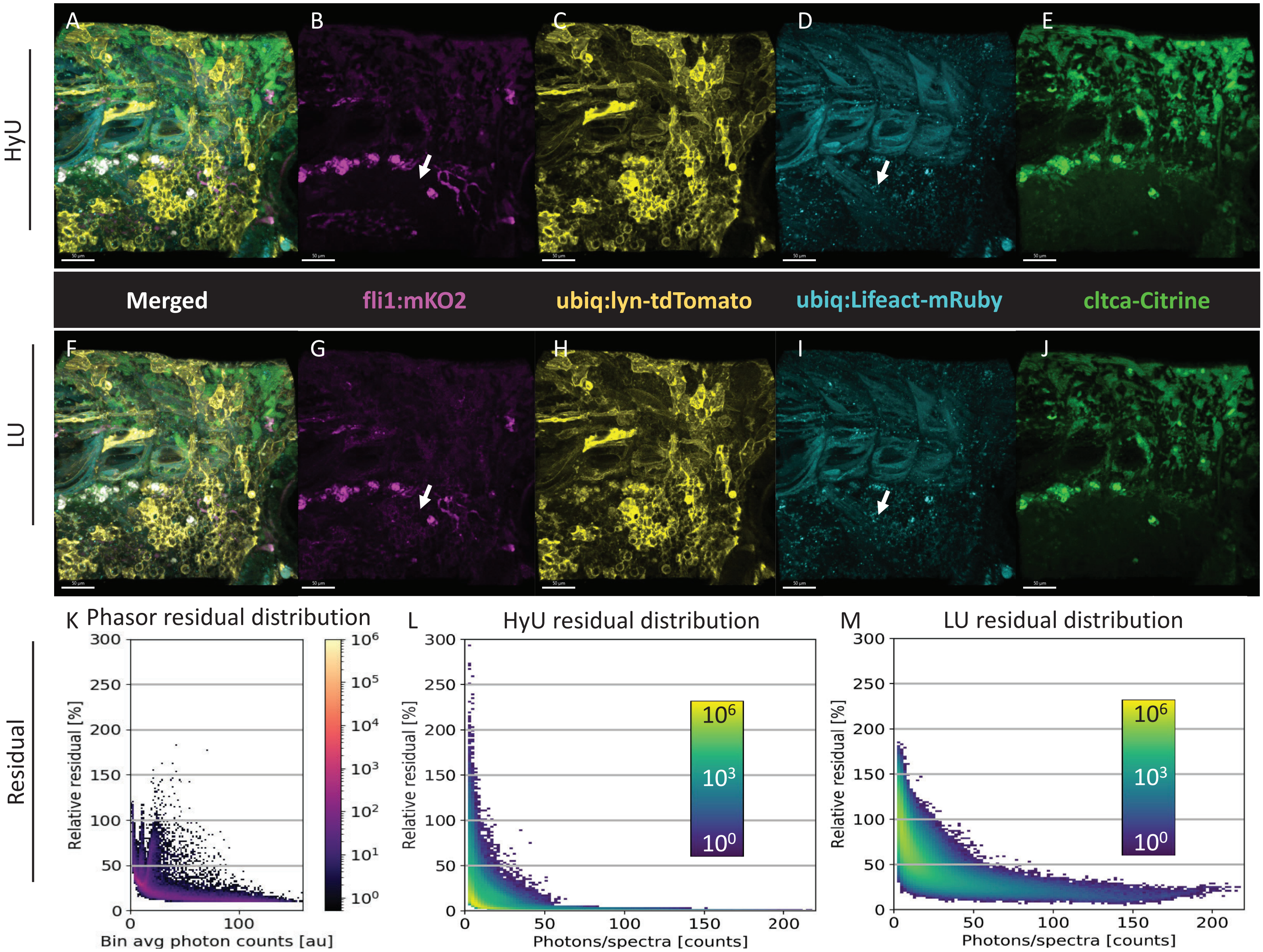

HyU
LU
Residual
A
B
C
D
E
Merged
fli1:mKO2
ubiq:lyn-tdTomato
ubiq:Lifeact-mRuby
cltca-Citrine
F
G
H
I
J
K
Phasor residual distribution
Relative residual [%]
Bin avg photon counts [au]
L
HyU residual distribution
Photons/spectra [counts]
M
LU residual distribution

Supplementary Figure 7. Unmixing of a quadra-transgenic zebrafish with HyU and LU highlights improvements in contrast and spatial features. Volumetric zoom-in view of the somites within the trunk region of a 10-dpf *Gt(cltca-citrine);Tg(ubiq:lyn-tdTomato;ubiq:Lifeact-mRuby;fli1:mKO2)* zebrafish merging all channels in **(A)** HyU and **(B)** LU. (**A-E**) HyU presents a wide dynamic range of intensities with average contrast 1.11-fold higher than LU compared to (**F-J**) LU. In LU, bleed-through from the **(H)** membrane label (arrow) is observed in the **(G)** lymphatic vasculature channel (arrow) and the **(I)** actin channel (arrow). This incorrect re-assignment of intensities is not present in the corresponding HyU channels for **(B)** vasculature and **(D)** actin, where fibers (arrow) are cleanly unmixed. **(K)** Phasor Residual Distribution shows the distribution of relative residual (%) and photon counts in phasor histogram bins. Residual distribution shows the distribution of relative residual (%) and photon counts in histogram pixels for both **(L)** HyU and **(M)** LU. The sample depicted is representative of 28 experimental sessions each with three to five biological replicates, yielding similar results.

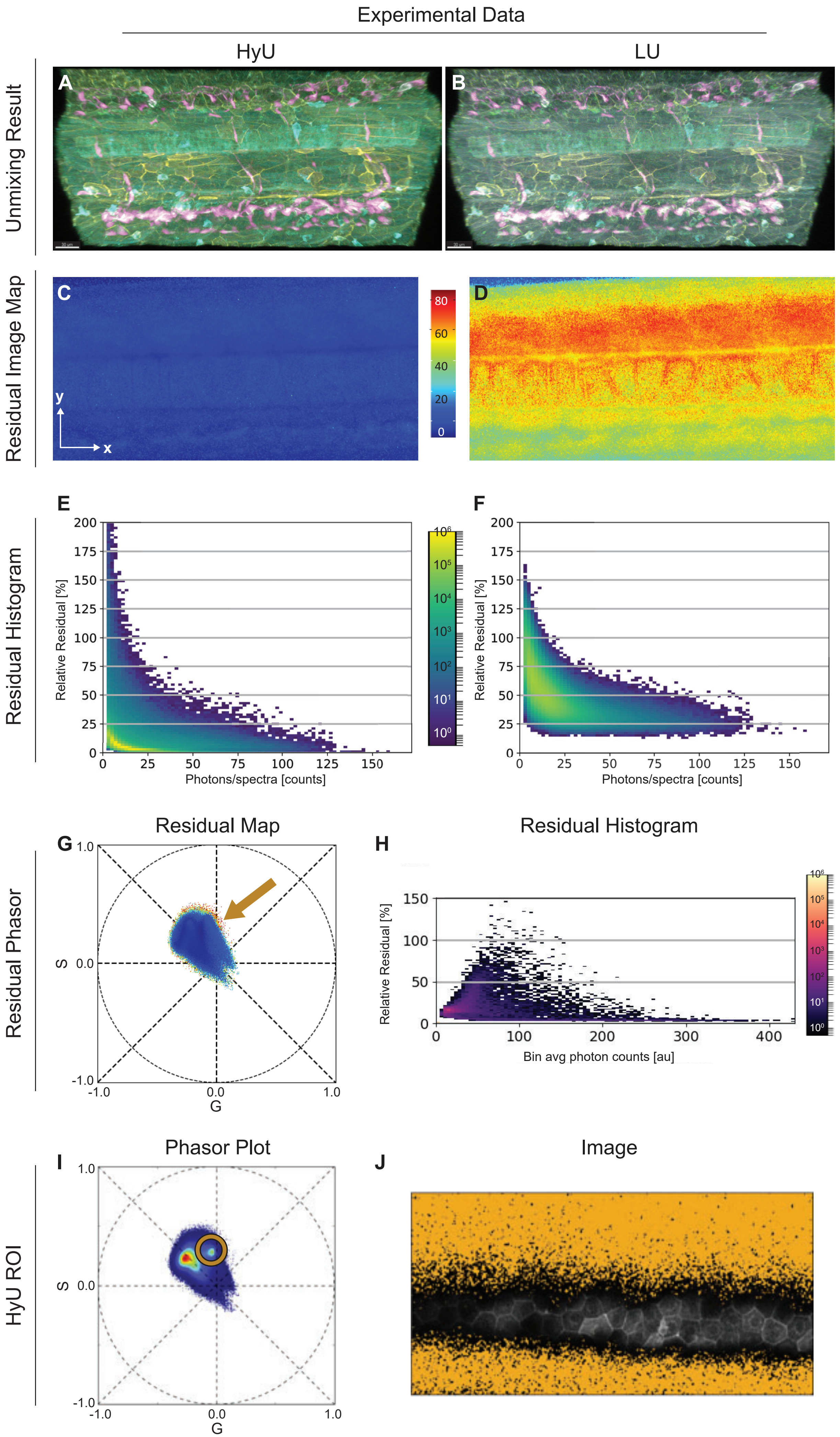

Experimental Data
HyU
LU
Unmixing Result
A
B
Residual Image Map
C
D
y
x
80
60
40
20
0
Residual Histogram
E
F
Relative Residual [%]
Photons/spectra [counts]
Residual Phasor
Residual Map
Residual Histogram
G
H
S
G
Bin avg photon counts [au]
HyU ROI
Phasor Plot
Image
I
J

Supplementary Figure 8. Residual analysis of experimental data supports performance improvement of HyU. Residual analysis for multispectral fluorescent data of a 5dpf quadra-transgenic zebrafish *Gt(cltca-citrine);Tg(ubiq:lyn-tdTomato;ubiq:Lifeact-mRuby;fli1:mKO2)* in Figure 3. Unmixing results for **(A)** HyU and **(B)** LU, respectively. Residual Image map of the z-averaged dataset for **(C)** HyU and **(D)** LU show lower residual values for HyU, suggesting improved quality of unmixing. **(E)** Residual distribution relative to the original intensity in each pixel as a function of estimated photon counts per spectrum for LU and (**F**) HyU. **(G)** Residual Phasor Map presents increased residual values in the background region (arrow). Jet colormap scale refers to **C**, **D** and **G**. **(H)** Residual Phasor Histogram for HyU shows distribution of residuals in the broad dynamic range of photons for experimental data. **(I)** Raw phasor with 0 threshold applied and 5 denoising filters, the ROI (yellow circle) highlights the background pixels in the **(J)** average spectral image (showing the first z slice).

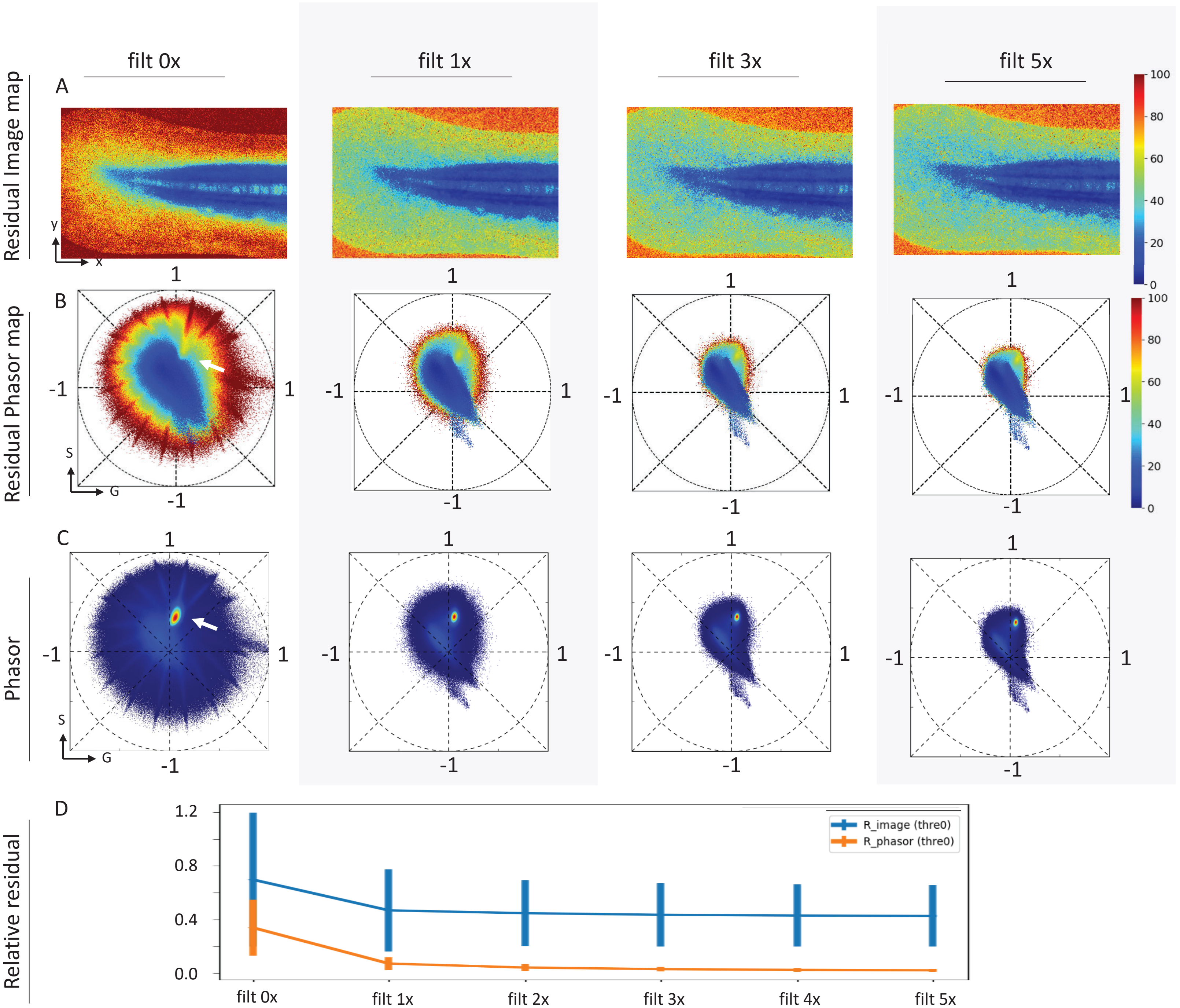

filt 0x
filt 1x
filt 3x
filt 5x
A
Residual Image map
B
Residual Phasor map
C
Phasor
D
Relative residual
R_image (thre0)
R_phasor (thre0)
filt 0x
filt 1x
filt 2x
filt 3x
filt 4x
filt 5x

Supplementary Figure 9. Application of denoising filters reveals improved results with lower residuals. (A) Residual Image Map of HyU unmixing of a quadra-transgenic zebrafish *Gt(cltca-citrine);Tg(ubiq:lyn-tdTomato;ubiq:Lifeact-mRuby;fli1:mKO2)* inclusive of one strong autofluorescent signal. Residual values are calculated with different number of denoising filters[1]. The average relative residual visibly decreases with the increase of denoising filter numbers. **(B)** Residual Phasor Map shows a major decrease in values between 0 and 1 denoising filters, maintaining statistically similar values at higher denoising filter applications. **(C)** Phasor plots for different denoising filters. Phasor of raw data prior to denoising or thresholding, presents noise connected to each of the detectors, as well as a high count area corresponding to the background region. The phasor plot distribution highlights areas with higher pixel counts coming from the background noise, which correspond with lower Residual Phasor Map values in B. **(D)** Average residual values for Residual Image Map **(A)** and Residual Phasor Map **(B)** highlight that the improvement on residuals mostly focuses on the first application of the denoising filter. In this initial denoising, the average relative residual decreased from 69.8% to 46.8%, further decreasing to 42.6% after 5 denoising. Average relative residual for phasor decreased from 33.9% to 7.1% after 1 denoising filter was applied, further decreasing to 2.1% after 5 denoising applied. With standard processing threshold of 250 digital levels applied (bottom 0.38% intensities of 16-bit format), the average relative residual decreased from 7.2% to 4.6%, further decreasing to 4.1% after 5 denoising filters. Average relative residual for phasor decreases from 10.1% to 2.6%, further decreasing to 1.1% after 5 denoising filters. Bars denote the variance of the relative residual values.

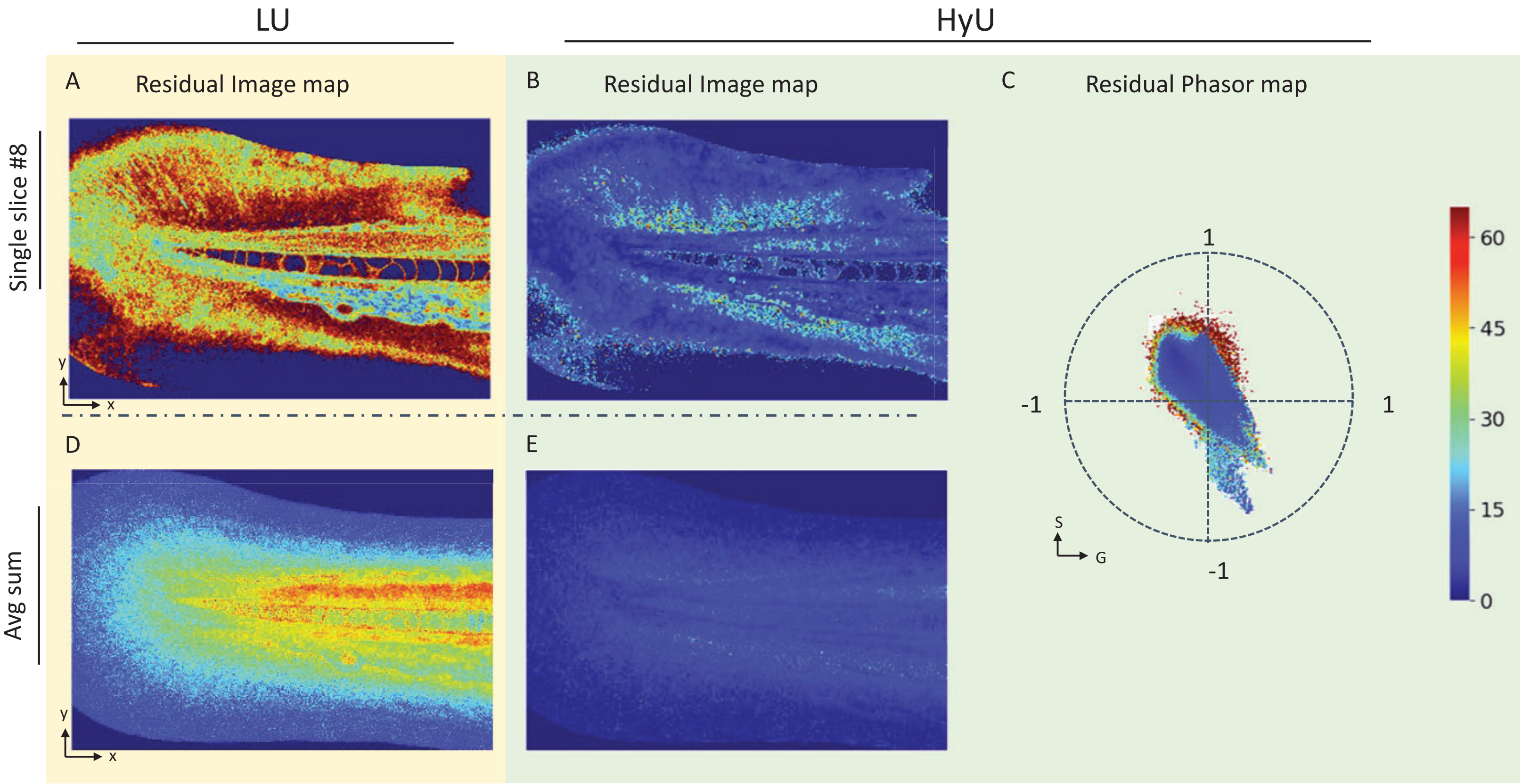

LU
HyU
A
Residual Image map
B
Residual Image map
C
Residual Phasor map
Single slice #8
D
E
Avg sum
y
x
S
G
1
-1
1
-1
60
45
30
15
0

Supplementary Figure 10. Comparison of residual images for LU and HyU highlights improved HyU performance. Residual Image projection for LU and HyU of a 3D dataset of 3 dfp quadra-transgenic zebrafish *Gt(cltca-citrine);Tg(ubiq:lyn-tdTomato; ubiq:Lifeact-mRuby;fli1:mKO2)* with an intensity threshold of 250. **(A)** LU Residual Image Map for a single slice (z=8 of 17 in a z-stack) provides average relative residual of 35.9%, while the **(B)** corresponding map for HyU averages at 7.1%. **(C)** Residual Phasor Map for the z-stack presents average relative residual of 1.1%. The reduction in residuals for HyU is maintained across the z-stack, as shown in **(D)** the average LU and **(E)** HyU Residual Image Maps built from the average of residuals across all z-slices. The average residual improvement for HyU at 4% compared to LU at 21% is 5.3-fold.

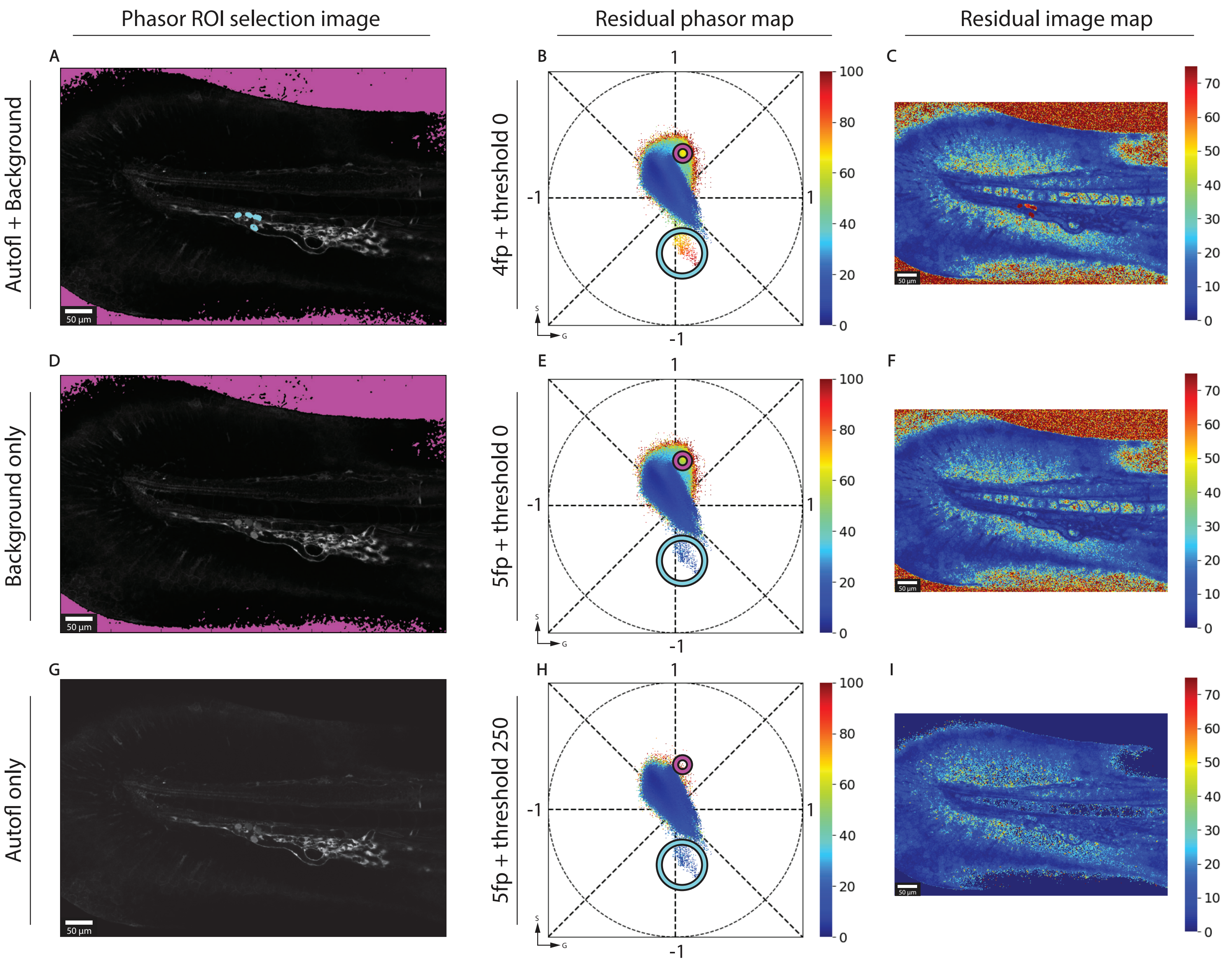

Phasor ROI selection image
Residual phasor map
Residual image map
A
B
C
D
E
F
G
H
I
Autofl + Background
Background only
Autofl only
4fp + threshold 0
5fp + threshold 0
5fp + threshold 250
50 µm
1
-1
S
G
100
80
60
40
20
0
70
50
30
10

Supplementary Figure 11. Residual maps facilitate identification of independent spectral components. Experimental fluorescence microscopy data often includes unexpected autofluorescence signals. Residual Maps (Methods) provide additional information to account for these signals and properly adjust HyU analysis. **(A)** Average intensity image with pixels pseudo-colored in cyan (autofluorescence) and magenta (background) according to the ROIs selections on the **(B)** Residual Phasor Map, computed from the HyU of 4 input spectra with a threshold of zero. The pseudo-colored areas of the image match those presenting high residual values in the **(C)** Residual Image Map. Changing the unmixing input to include the unexpected autofluorescent spectrum (cyan) and performing the Residual Phasor map selections produces the **(D)** background pseudo-colored (magenta) image. The inclusion of autofluorescence as an independent spectral component in the unmixing decreases the number of pixels corresponding to the autofluorescent signal (cyan ROI) in the **(E)** Residual Phasor Map, thereby matching with the **(F)** Residual Image Map, which no longer presents high residuals in the center portion of the image. Increasing the threshold to 250 removes the pixels with high residuals corresponding to the background, removing them from the **(G)** average intensity image, **(H)** Residual Phasor Map, and **(I)** Residual Image Map.

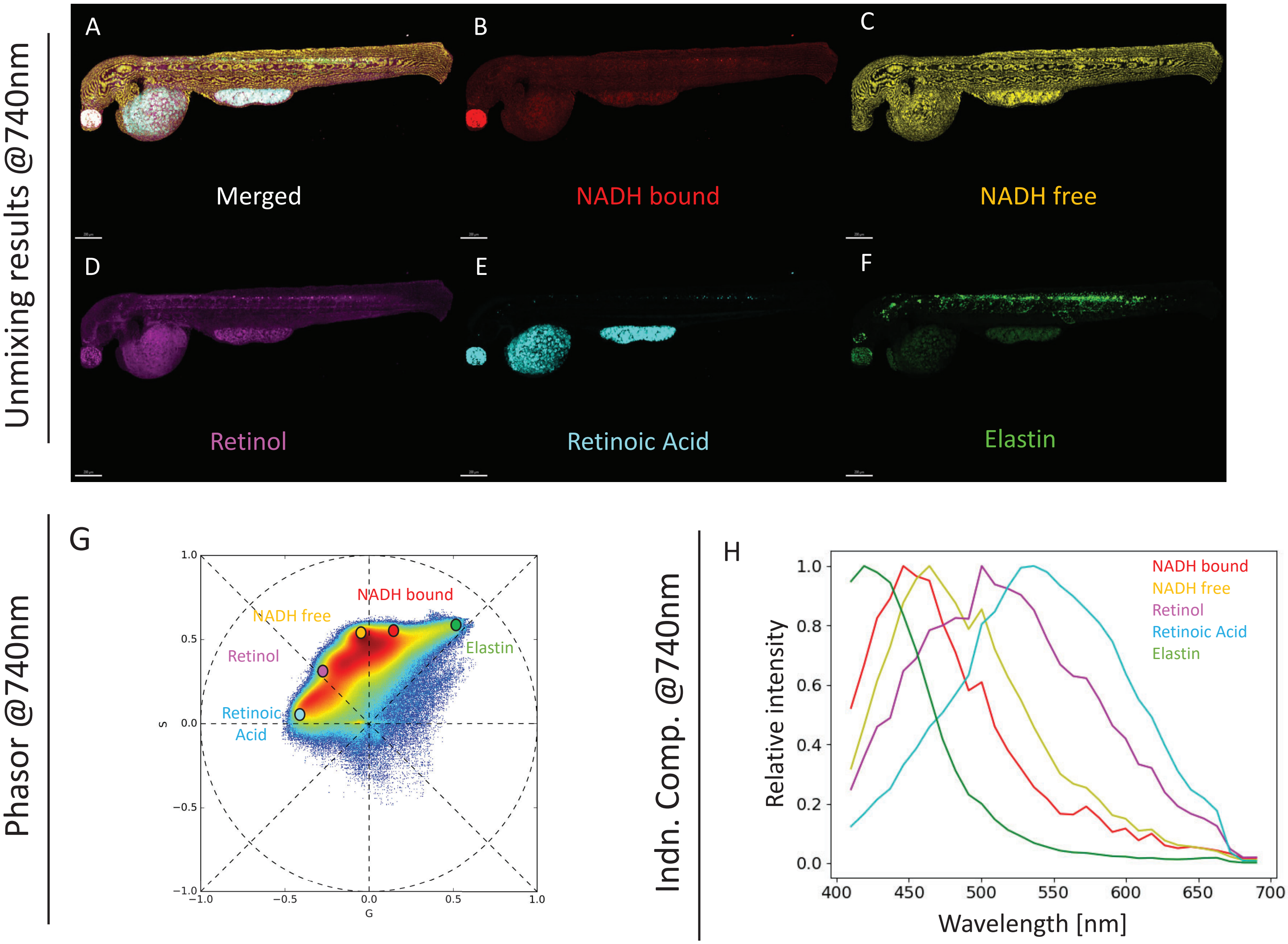

Unmixing results @740nm
A
Merged
B
NADH bound
C
NADH free
D
Retinol
E
Retinoic Acid
F
Elastin
Phasor @740nm
G
NADH bound
NADH free
Retinol
Elastin
Retinoic
Acid
S
G
Indn. Comp. @740nm
H
NADH bound
NADH free
Retinol
Retinoic Acid
Elastin
Relative intensity
Wavelength [nm]

Supplementary Figure 12. HyU analysis of 36 hpf Casper zebrafish demonstrates feasibility of unmixing only intrinsic signals. Casper zebrafish is a transgenic zebrafish characterized by the absence of pigments. Dataset was acquired in the two-photon spectral mode @740 nm excitation. HyU Unmixing was performed utilizing 5 pure intrinsic signals measured in solution (Methods): **(A)** Merged overview of all signals **(B)** NADH bound **(C)** NADH free (yellow), **(D)** retinoid (magenta), **(E)** retinoic acid (cyan) which appears mainly in the yolk sac, known location where carotenoids are stored, transferred and then metabolized to retinoic acid [2] **(F)** elastin (green) has a similar distribution with in the zebrafish floorplate at this developmental stage **(G)** Phasor **(H)** average spectra from selection in **G**. The sample depicted is representative of 24 experimental sessions each with five biological replicates, yielding similar results.

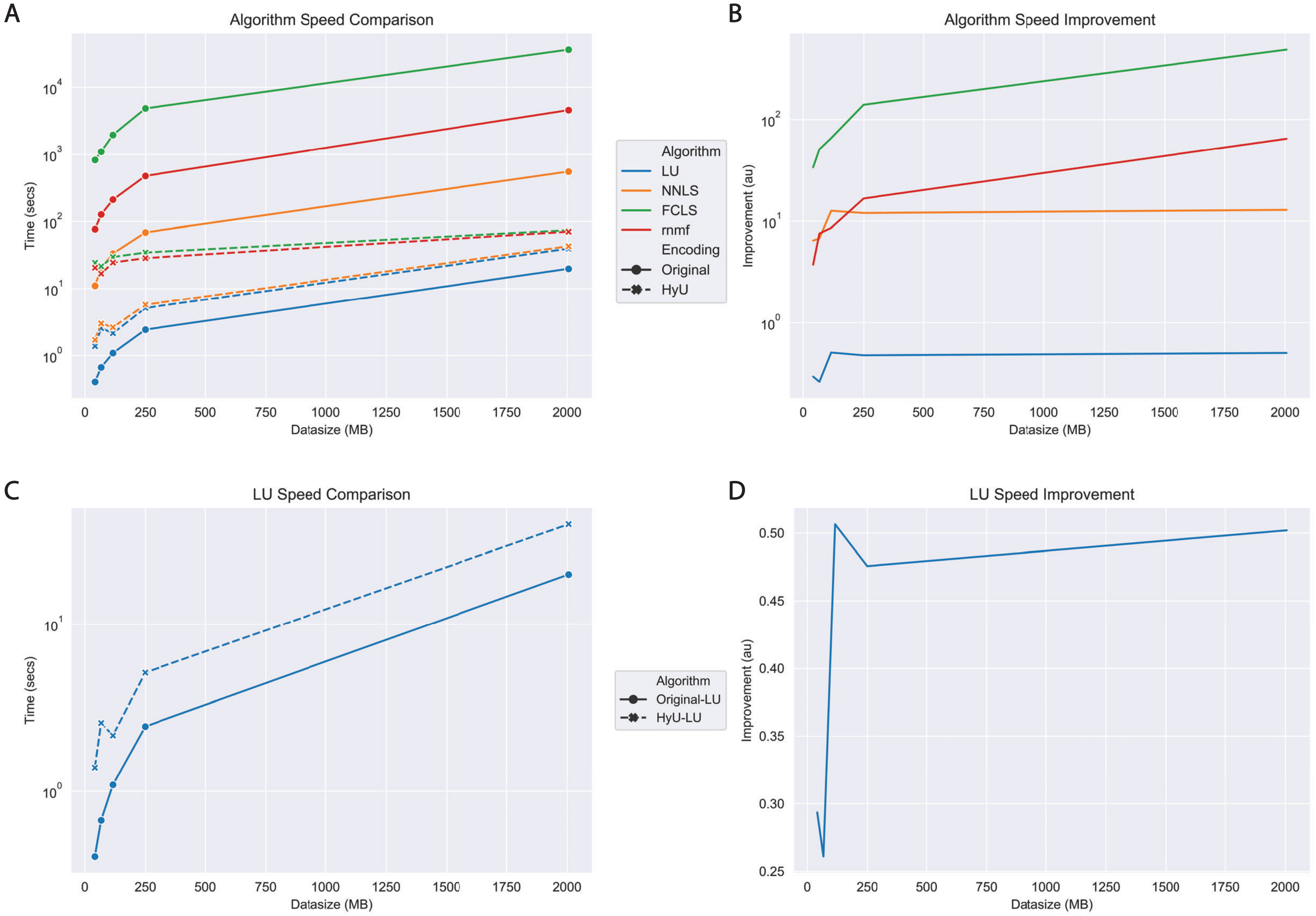

A
Algorithm Speed Comparison
Time (secs)
Datasize (MB)
B
Algorithm Speed Improvement
Improvement (au)
Datasize (MB)
Algorithm
LU
NNLS
FCLS
rnmf
Encoding
Original
HyU
C
LU Speed Comparison
Time (secs)
Datasize (MB)
Algorithm
Original-LU
HyU-LU
D
LU Speed Improvement
Improvement (au)
Datasize (MB)

Supplementary Figure 13. Speed comparison and improvement plots of multiple unmixing algorithms in their Original form vs HyU encoded. **(A)** Computational times of multiple unmixing algorithms for both original (pixel-by-pixel) and HyU versions over a range of hyperspectral imaging datasets sizes. **(B)** Improvement in speed using the ratio of the HyU over the Original unmixings demonstrate a vast increase in speed for all algorithms other than LU across all input data sizes. **(C, D)** Computational times and speed improvement for original and HyU versions of LU show that the original version of LU provides higher computational speeds at ~2x. Plots **A-C** use logarithmic scales while plot **D** uses a linear scale for the y-axis.

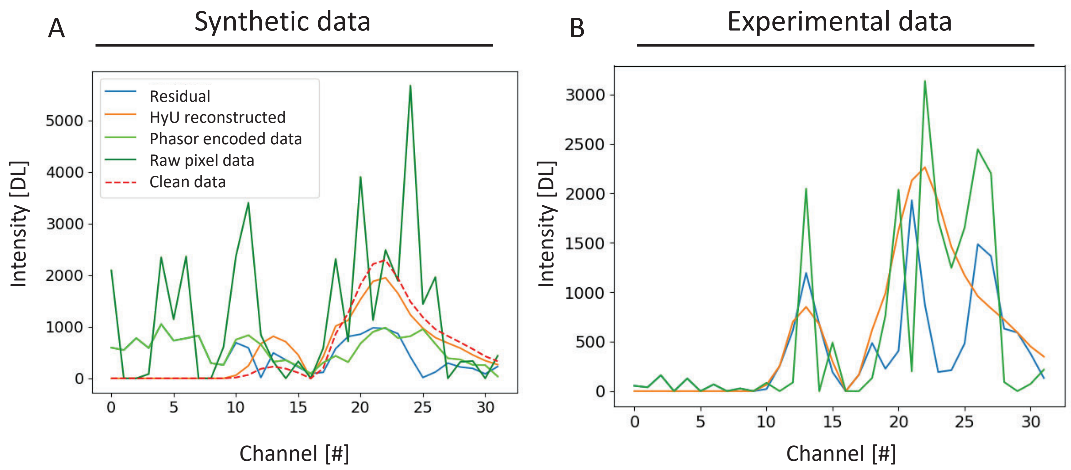
A
Synthetic data
B
Experimental data
Residual
HyU reconstructed
Phasor encoded data
Raw pixel data
Clean data
Intensity [DL]
Channel [#]
0
5
10
15
20
25
30
1000
2000
3000
4000
5000
500
1500
2500

Supplementary Figure 14. Residuals in synthetic data and experimental data. (A) We simulated data with the purpose to cover wide ranges of noise and to allow for thorough testing of the algorithm's performance. In this example a ground truth spectrum with 14 photons (red dashed line) is simulated accounting for multiple types of noise (dark green line). The simulated spectrum presents disrupted shape with substantial presence of noise. During HyU analysis, in the encoding of spectra within a phasor bin, the simulated spectrum (dark green line) is averaged with similar spectra of multiple other pixels, producing (light green light). The spectrum recovered with Hybrid Unmixing (orange line) is similar to the ground truth. In the calculation of residuals, however, due to the disrupted signal of simulation (dark green line), the absence of noise in the clean data is counted as residual. **(B)** Spectrum from experimental data (from Figure 3) at a similar photon-range (15 to 20 photons) for comparison. The same color code is utilized for the spectra lines, without ground truth owing to the nature of experimental data.

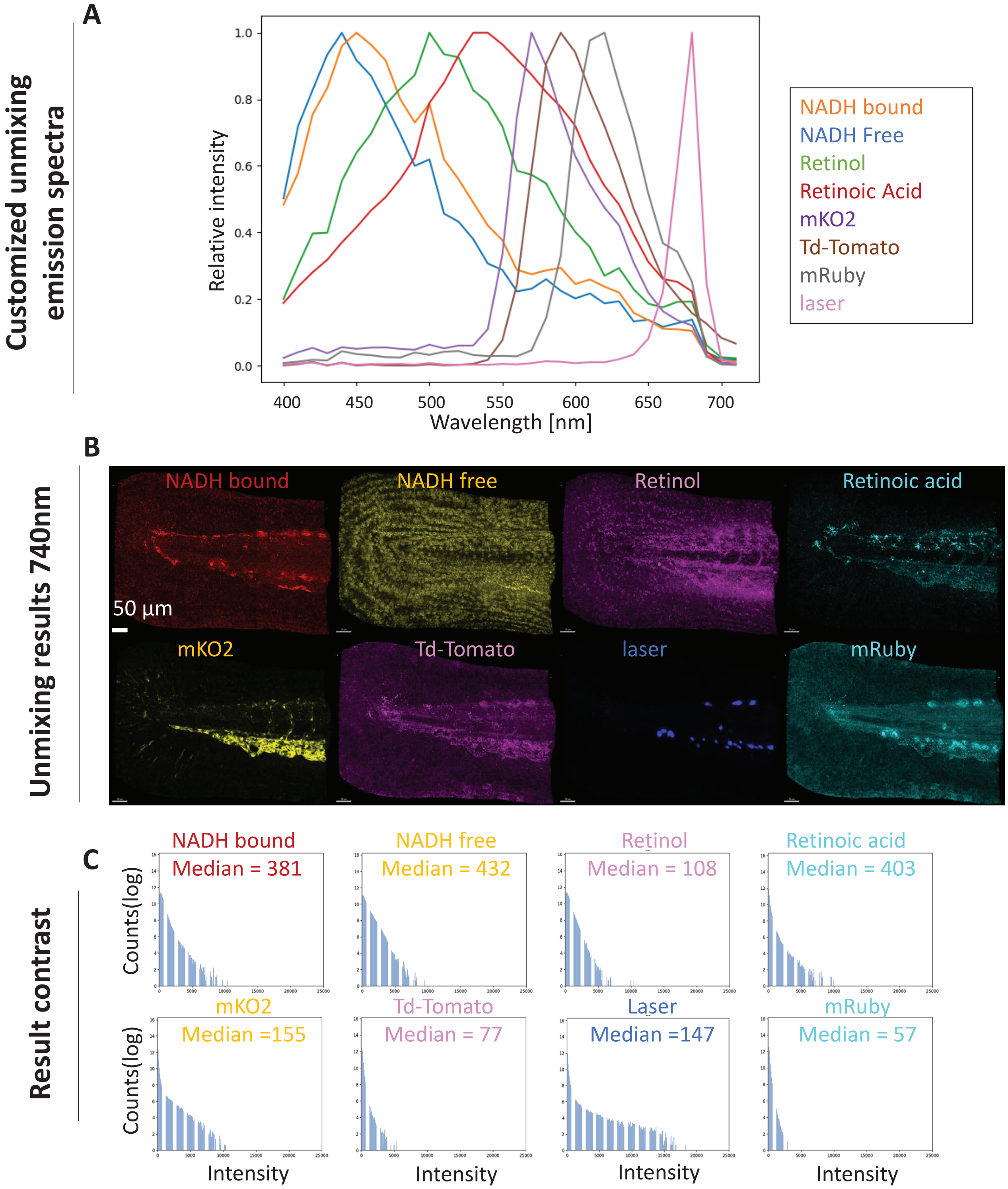
A
Customized unmixing emission spectra
Relative intensity
Wavelength [nm]
NADH bound
NADH Free
Retinol
Retinoic Acid
mKO2
Td-Tomato
mRuby
laser
B
Unmixing results 740nm
NADH bound
NADH free
Retinol
Retinoic acid
50 µm
mKO2
Td-Tomato
laser
mRuby
C
Result contrast
NADH bound
Median = 381
NADH free
Median = 432
Retinol
Median = 108
Retinoic acid
Median = 403
mKO2
Median =155
Td-Tomato
Median = 77
Laser
Median =147
mRuby
Median = 57
Counts(log)
Intensity

Supplementary Figure 15. HyU unmixing on low concentration signals using customized independent spectra

Results from unmixing intrinsic and extrinsic signals of a quadra-transgenic zebrafish: *Gt(cltca-citrine);Tg(ubiq:lyn-tdTomato; ubiq:Lifeact-mRuby;fli1:mKO2)* at a single timepoint of the dataset presented in Figure 6 provide further information and highlight the weak expression of some extrinsic signals in this dataset. **(A)** Input spectra for the intrinsic signatures were directly acquired by selection of the endmembers in the phasor plot. Input spectra for the extrinsic signatures were acquired from other datasets of samples expressing those signatures individually and excited at 740 nm 2-photon, since these extrinsic signals are not strongly expressed within this dataset. **(B)** Renderings of unmixing results were automatically adjusted to show the best contrast. Unmixing can still be performed with spectra from weak input signatures. (**C**) Histogram counts of each unmixed independent spectral signature demonstrate the low signals of the extrinsic fluorescence signatures compared to the intrinsic ones. The median value of the mRuby and tdTomato channels are 57 and 77 Digital Levels respectively, considerably lower than those of the other signals. The sample depicted is representative of 28 experimental sessions each with three to five biological replicates, yielding similar results.

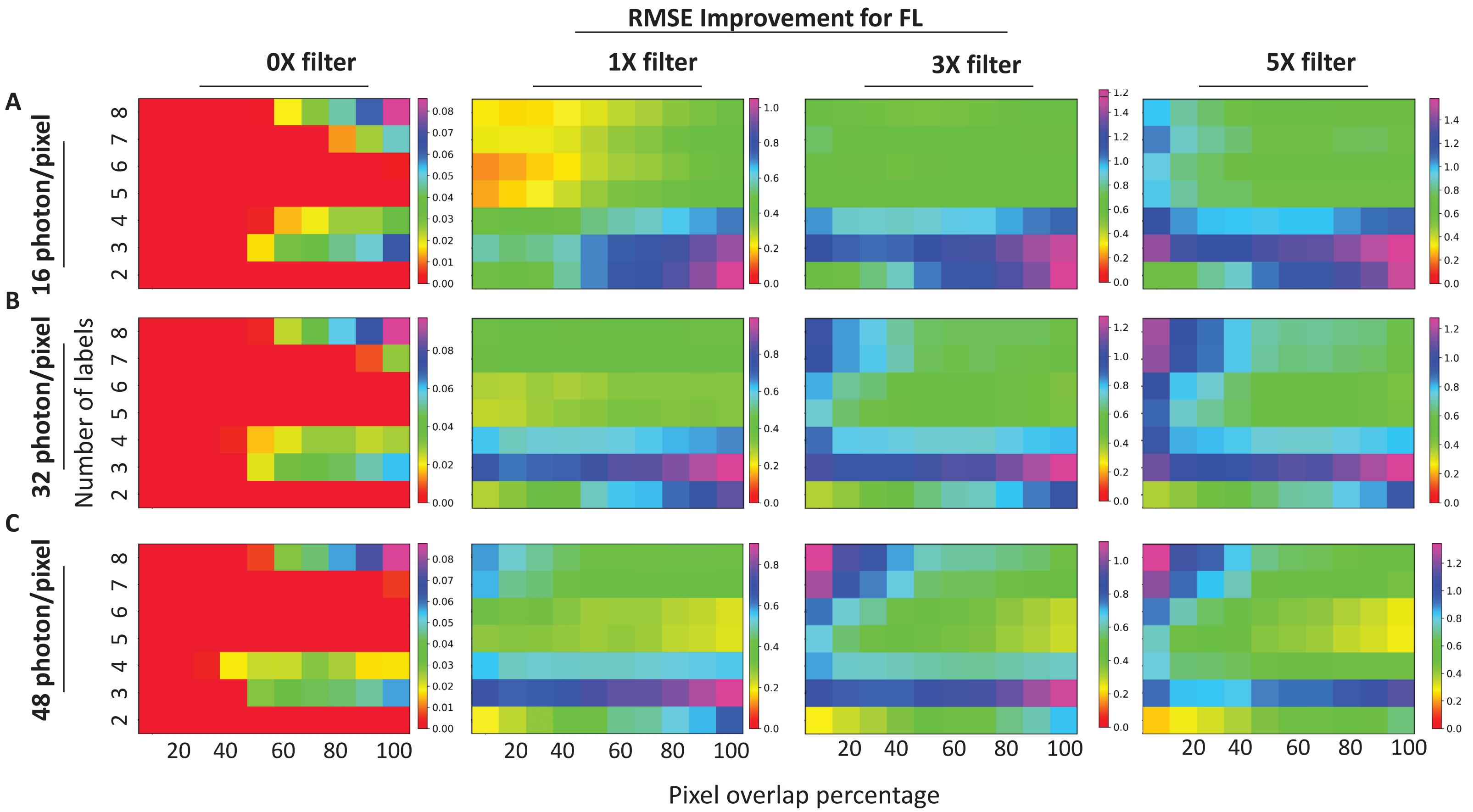

RMSE Improvement for FL
0X filter
1X filter
3X filter
5X filter
A
B
C
16 photon/pixel
32 photon/pixel
48 photon/pixel
Number of labels
Pixel overlap percentage

Supplementary Figure 16. RMSE improvement for simulated fluorescent spectral combinations highlights increased HyU performance across multiple denoising filters. Twelve matrices demonstrate the RMSE improvement of HyU with respect to LU when unmixing a collection of synthetic data with 2 to 8 extrinsic labels (Y axis of each matrix) as a function of the spatial overlap of these labels in a sample (X axis of each matrix). In the matrix, 0% overlap denotes simulations with spatially distinct fluorophores, where each pixel corresponds to a single fluorophore, while simulations with 100% overlap contain, in every pixel, a randomized ratio of the n fluorophores. Each one of the values reported in a matrix is the average of a 1024x1024x32 pixels simulation and shows the RMSE improvement of HyU to LU. Different columns in the figure report the RMSE improvement matrices with different numbers of denoising filters (0x, 1x, 3x, 5x) applied with a total number of photons per pixel at **(A)** 16 **(B)** 32 **(C)** 48. In the absence of denoising filters, the improvement of HyU overall is less than 8%. Denoising filters improve RMSE by over 80%. Spectra utilized for this simulation are reported in Sup. Figure 20A.

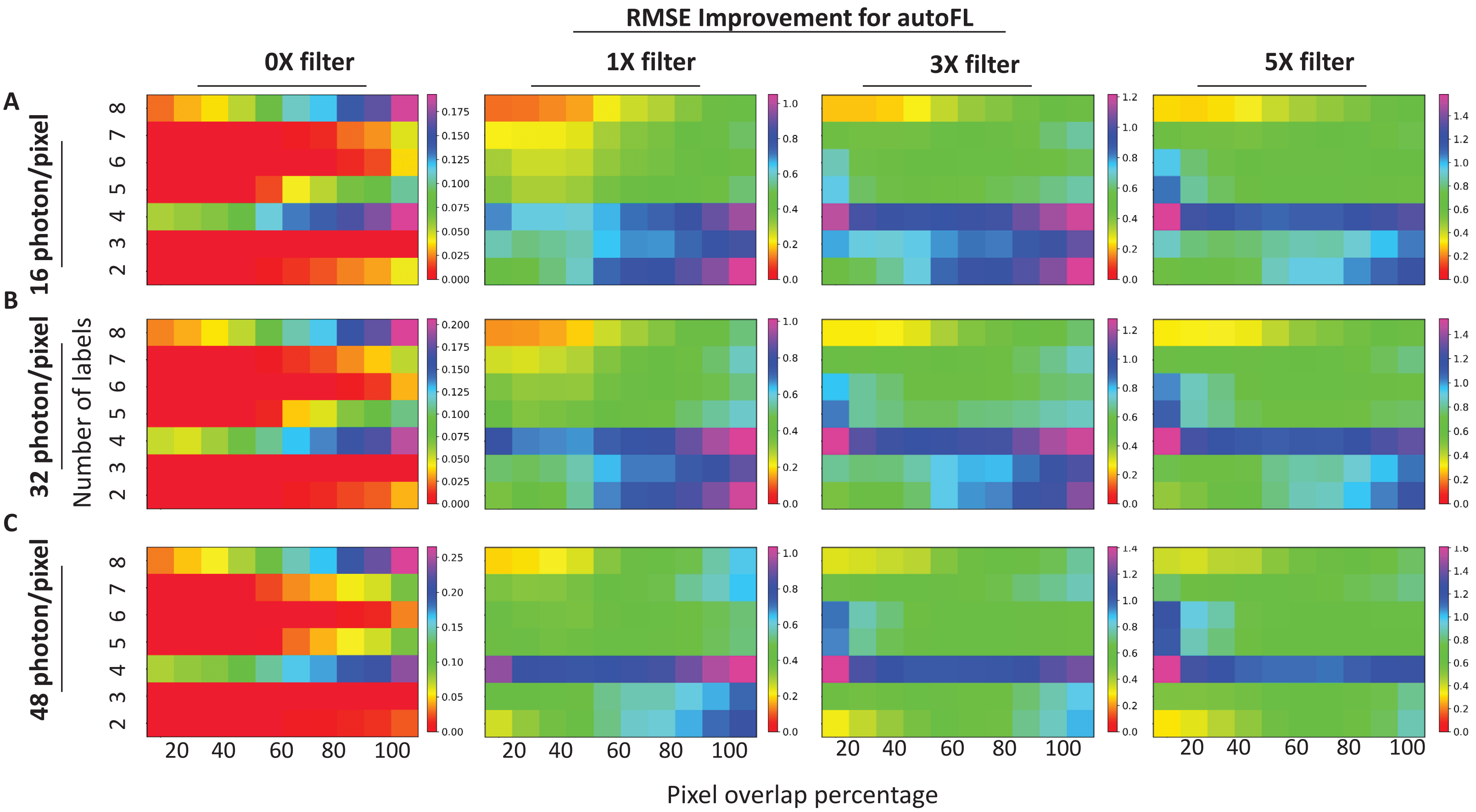

RMSE Improvement for autoFL
0X filter
1X filter
3X filter
5X filter
A
B
C
16 photon/pixel
32 photon/pixel
48 photon/pixel
Number of labels
Pixel overlap percentage

Supplementary Figure 17. RMSE improvement for simulated fluorescent and autofluorescent spectral combinations highlights increased HyU performance across multiple denoising filters

Twelve matrices demonstrate the RMSE improvement of HyU with respect to LU when unmixing a collection of synthetic data with 2 to 8 extrinsic and intrinsic labels (Y axis of each matrix) as a function of the spatial overlap of these labels in a sample (X axis of each matrix). In the matrix, 0% overlap denotes simulations with spatially distinct fluorophores, where each pixel corresponds to a single fluorophore, while simulations with 100% overlap have, in every pixel, a randomized ratio of the n extrinsic and intrinsic fluorophores. Each one of the values reported in a matrix is the average of a 1024x1024x32 pixels simulation and shows the RMSE improvement of HyU to LU. Different columns in the figure report the RMSE improvement matrices with different numbers of denoising filters (0x, 1x, 3x, 5x) applied with a total number of photons per pixel at **(A)** 16 **(B)** 32 **(C)** 48. In the absence of denoising filters, the improvement of HyU overall is less than 25%. Denoising filters improve RMSE by over 100%. Spectra utilized for this simulation are reported in Sup. Figure 20B.

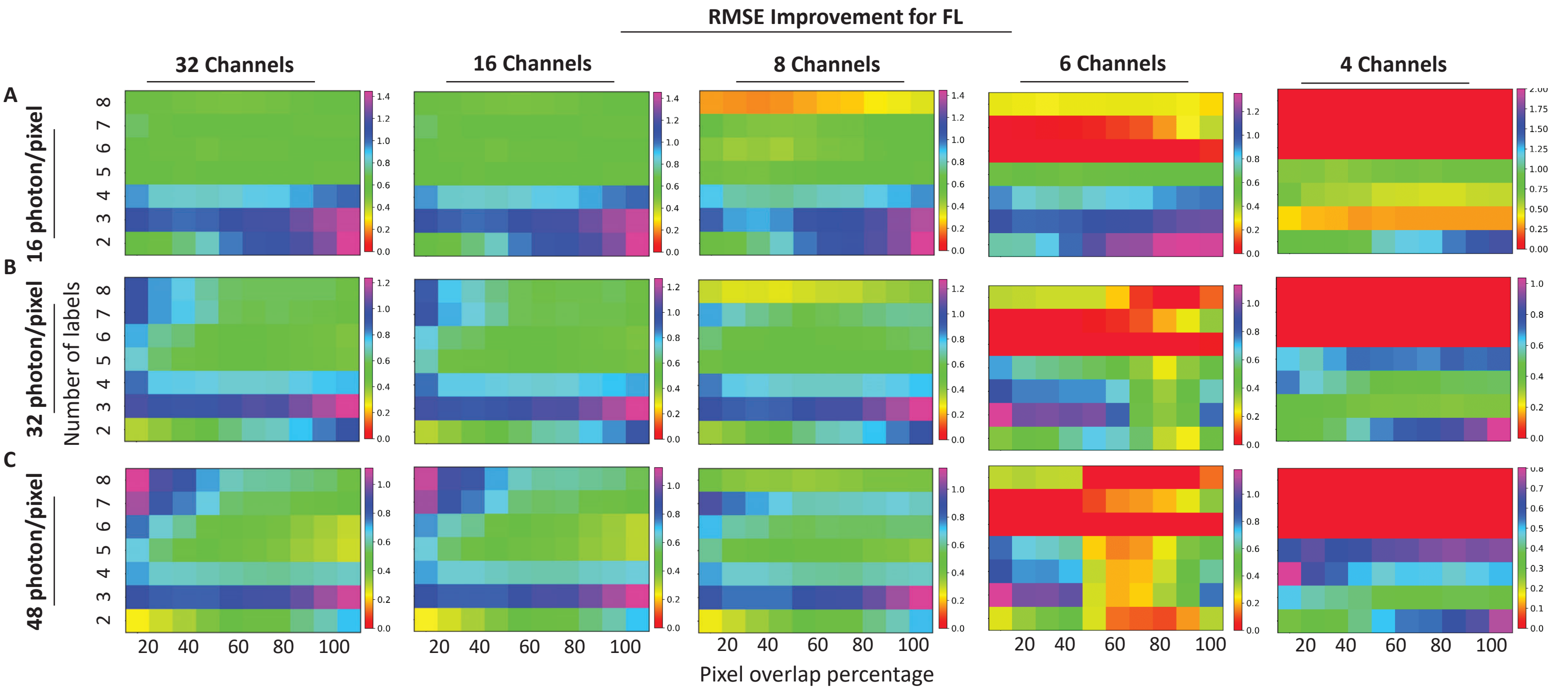
RMSE Improvement for FL
32 Channels
16 Channels
8 Channels
6 Channels
4 Channels
A
B
C
16 photon/pixel
32 photon/pixel
48 photon/pixel
Number of labels
Pixel overlap percentage

Supplementary Figure 18. RMSE improvement for simulated fluorescent spectral combinations highlights decreasing overall performance across decreased number of spectral channels

Fifteen matrices demonstrate the RMSE improvement of HyU with respect to LU when unmixing a collection of synthetic data with 2 to 8 extrinsic labels (Y axis of each matrix) as a function of the spatial overlap of these labels in a sample (X axis of each matrix). In the matrix, 0% overlap denotes simulations with spatially distinct fluorophores, where each pixel corresponds to a single fluorophore, while simulations with 100% overlap contain, in every pixel, a randomized ratio of the n fluorophores. Each one of the values reported in a matrix is the average of a 1024x1024x32 pixels simulation and shows the RMSE improvement of HyU to LU with 3x denoising filters. Columns in the figure represent RMSE improvement matrices across an increasingly binned number of spectral channels (32, 16, 8, 6, 4) applied with a total number of photons per pixel at **(A)** 16 **(B)** 32 **(C)** 48. When utilizing 32 spectral channels data, RMSE improvements reach above the previously reported 80% for highly overlapping fluorophores. Successively increasing the binning across the wavelength dimension (and therefore decreasing the number of spectral channels) shows a slow downward trend of the RMSE improvement until the 4 spectral channels matrices, where the RMSE improvement drops drastically down to below 8%, especially for more than 6 labels. Spectra utilized for this simulation are reported in Sup. Figure 20A.

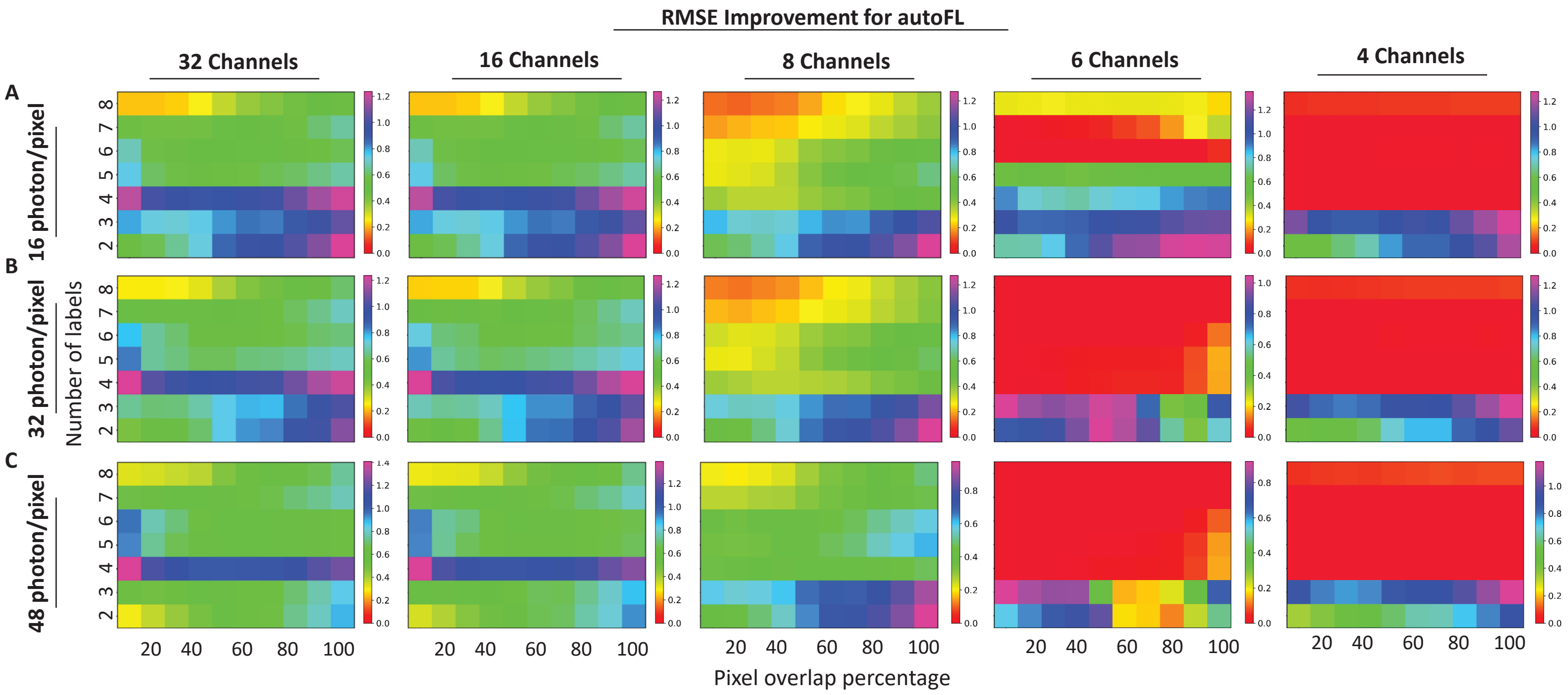

RMSE Improvement for autoFL
32 Channels
16 Channels
8 Channels
6 Channels
4 Channels
A
B
C
16 photon/pixel
32 photon/pixel
48 photon/pixel
Number of labels
Pixel overlap percentage

Supplementary Figure 19. RMSE improvement for simulated fluorescent and autofluorescent spectral combinations highlights decreasing overall performance across decreased number of spectral channels

Fifteen matrices demonstrate the RMSE improvement of HyU with respect to LU when unmixing a collection of synthetic data with 2 to 8 extrinsic and intrinsic labels (Y axis of each matrix) as a function of the spatial overlap of these labels in a sample (X axis of each matrix). In the matrix, 0% overlap denotes simulations with spatially distinct fluorophores, where each pixel corresponds to a single fluorophore, while simulations with 100% overlap contain, in every pixel, a randomized ratio of the n extrinsic and intrinsic fluorophores. Each one of the values reported in a matrix is the average of a 1024x1024x32 pixels simulation and shows the RMSE improvement of HyU to LU. Columns in the figure represent RMSE improvement matrices with 3x denoising filters across an increasingly binned number of spectral channels (32, 16, 8, 6, 4) applied with a total number of photons per pixel at **(A)** 16 **(B)** 32 **(C)** 48. When utilizing 32 spectral channel data, RMSE improvements reach up to the previously reported 100% for highly overlapping fluorophores. Successively increasing the binning across the wavelength dimension (and therefore decreasing the number of channels) shows a slow downward trend of the RMSE improvement until the 4 spectral channel matrices, where the RMSE improvement drops drastically down to below 25%, especially for more than 3 labels. Spectra utilized for this simulation are reported in Sup. Figure 20B.

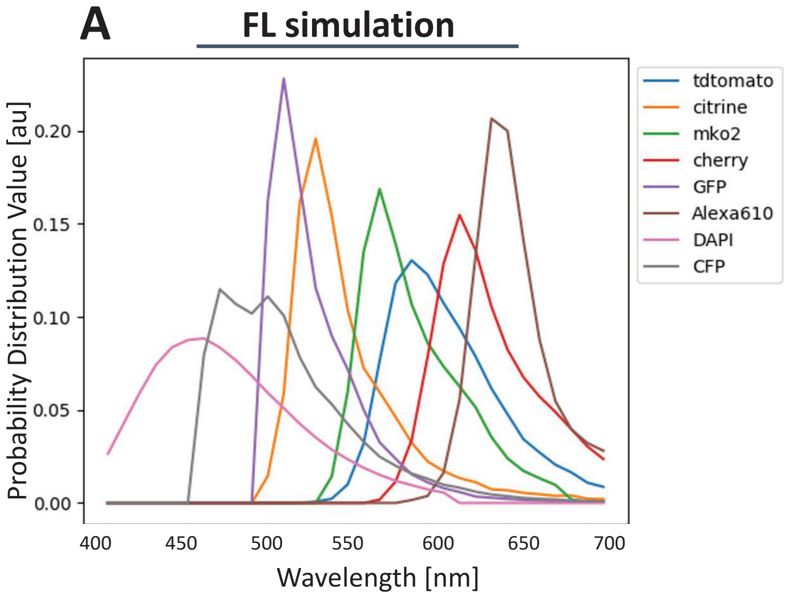

A
FL simulation
Probability Distribution Value [au]
0.00
0.05
0.10
0.15
0.20
400
450
500
550
600
650
700
Wavelength [nm]
tdtomato
citrine
mko2
cherry
GFP
Alexa610
DAPI
CFP

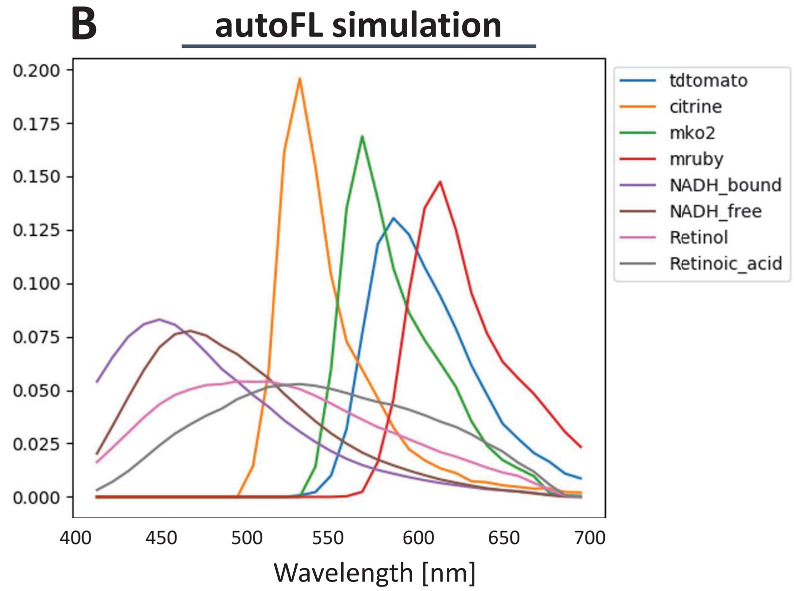

B
autoFL simulation
0.000
0.025
0.050
0.075
0.100
0.125
0.150
0.175
0.200
400
450
500
550
600
650
700
Wavelength [nm]
tdtomato
citrine
mko2
mruby
NADH_bound
NADH_free
Retinol
Retinoic_acid

Supplementary Figure 20. Emission spectra of components in overlapping simulation
(A) Emission spectra from 8 fluorophores including tdTomato, Citrine, mKO2, mCherry, GFP, Alexa610, DAPI, and CFP used for Fluorescence (FL) overlapping simulations. **(B)** Emission spectra from 8 extrinsic and intrinsic fluorophores including tdTomato, Citrine, mKO2, mRuby, NADH bound, NADH free, Retinol, and Retinoic acid used for autofluorescence (autoFL) overlapping simulations.

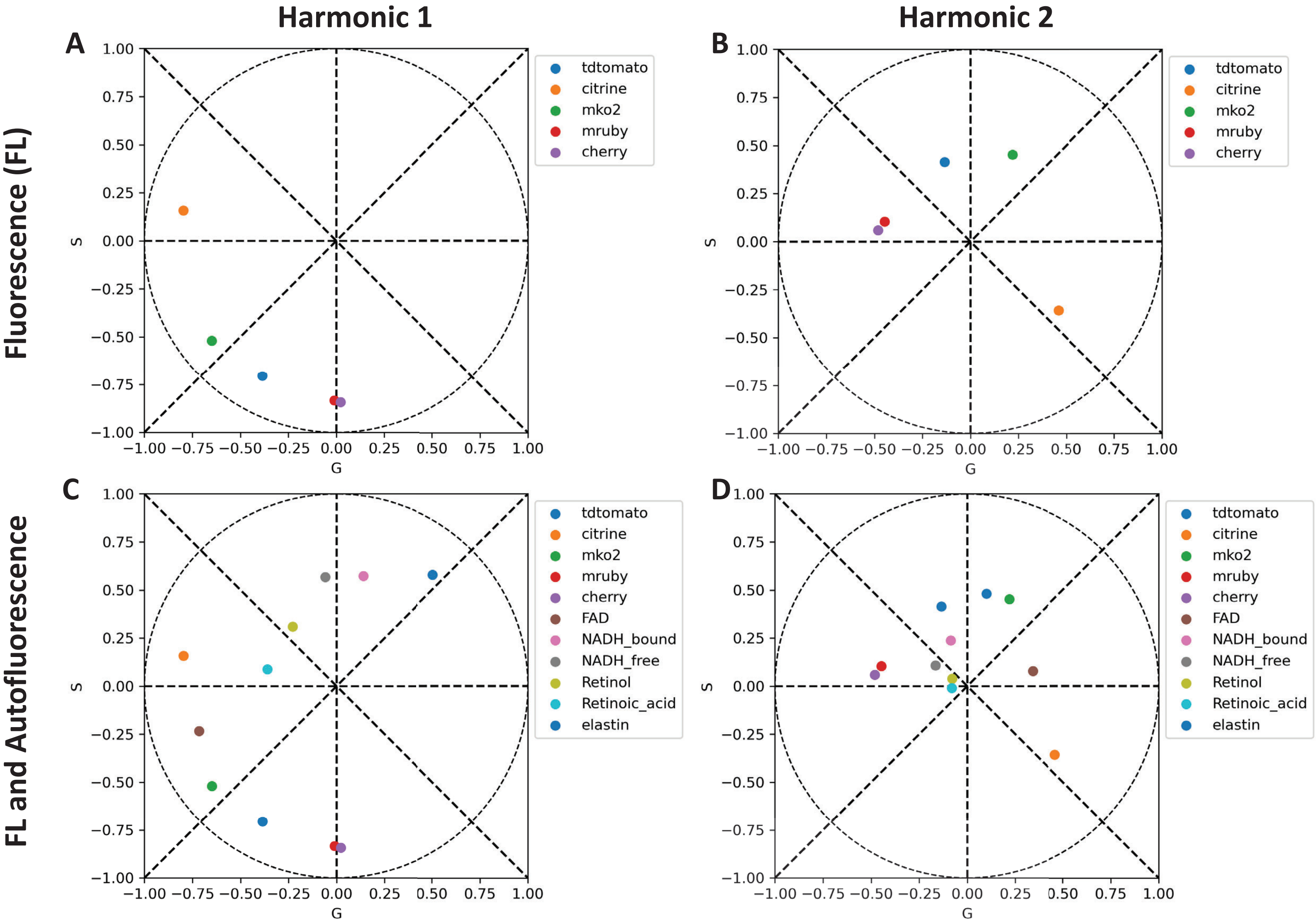

Harmonic 1
Harmonic 2
Fluorescence (FL)
FL and Autofluorescence
A
B
C
D
S
G
tdtomato
citrine
mko2
mruby
cherry
FAD
NADH_bound
NADH_free
Retinol
Retinoic_acid
elastin
1.00
0.75
0.50
0.25
0.00
−0.25
−0.50
−0.75
−1.00

Supplementary Figure 21. Pre-identified positions for common fluorophores on the phasor map

(A) Pre-identified extrinsic label positions (g,s) are denoted on the phasor plot for the first harmonic and **(B)** second harmonic. **(C)** Intrinsic label locations are further added on the phasor plot for the first harmonic and **(D)** second harmonic. Second harmonic generally covers a larger portion of the phasor space compared to the first harmonic. However, in the case of intrinsic signals, the locations of the pure autofluorescence spectra are on average more separated when utilizing the first harmonic. Details on the source of the pure spectra for these locations are reported in Methods – Independent Spectral Signatures.

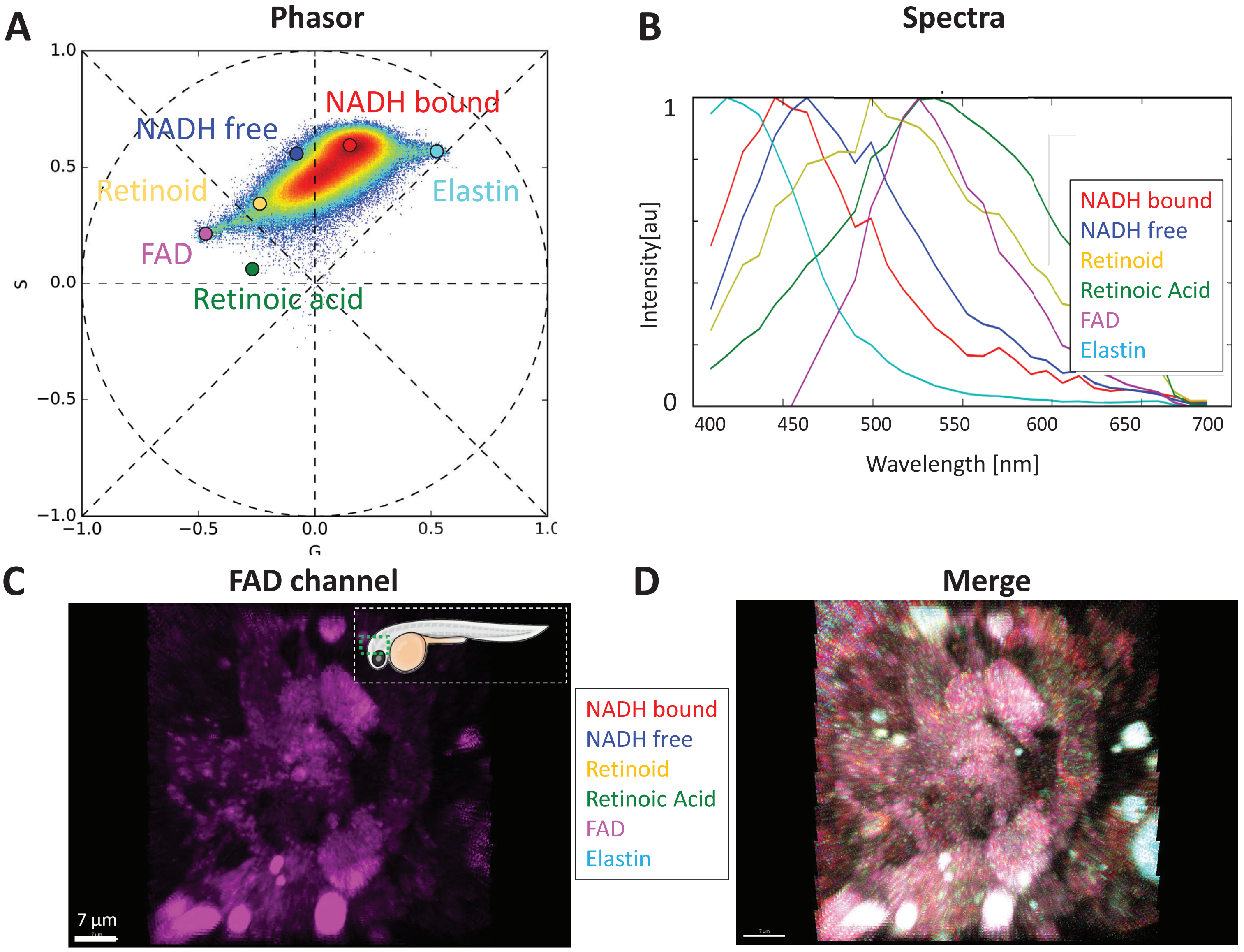
A
Phasor
NADH bound
NADH free
Retinoid
Elastin
FAD
Retinoic acid
1.0
0.5
0.0
−0.5
−1.0
S
G
B
Spectra
Intensity[au]
Wavelength [nm]
400
450
500
550
600
650
700
NADH bound
NADH free
Retinoid
Retinoic Acid
FAD
Elastin
C
FAD channel
7 µm
NADH bound
NADH free
Retinoid
Retinoic Acid
FAD
Elastin
D
Merge

Supplementary Figure 22. FAD autofluorescence in high magnification brain region of zebrafish embryo

(A) Phasor analysis reveals a distinct autofluorescence spectral component (magenta dot) when utilizing 740 nm 2-photon excitation to image a 22 hpf wild type zebrafish brain with high magnification (pixel size = 0.078x0.078µm) and high power (Table S1). **(B)** The corresponding emission spectra from the phasor selections in **A**. The spectrum corresponding to the magenta phasor selection in **A** closely matches the spectral signal of FAD obtained from in vitro solutions (Methods – Independent Spectral Signatures) and accounting for local environment changes[3]. **(C)** FAD unmixing channel highlights the FAD cluster in the head region of zebrafish. **(D)** Composite image rendering of the unmixing results for the intrinsic signals: NADH bound, NADH free, Retinoid, Retinoic Acid, FAD, and Elastin. The sample depicted is representative of 24 experimental sessions each with five biological replicates, yielding similar results.

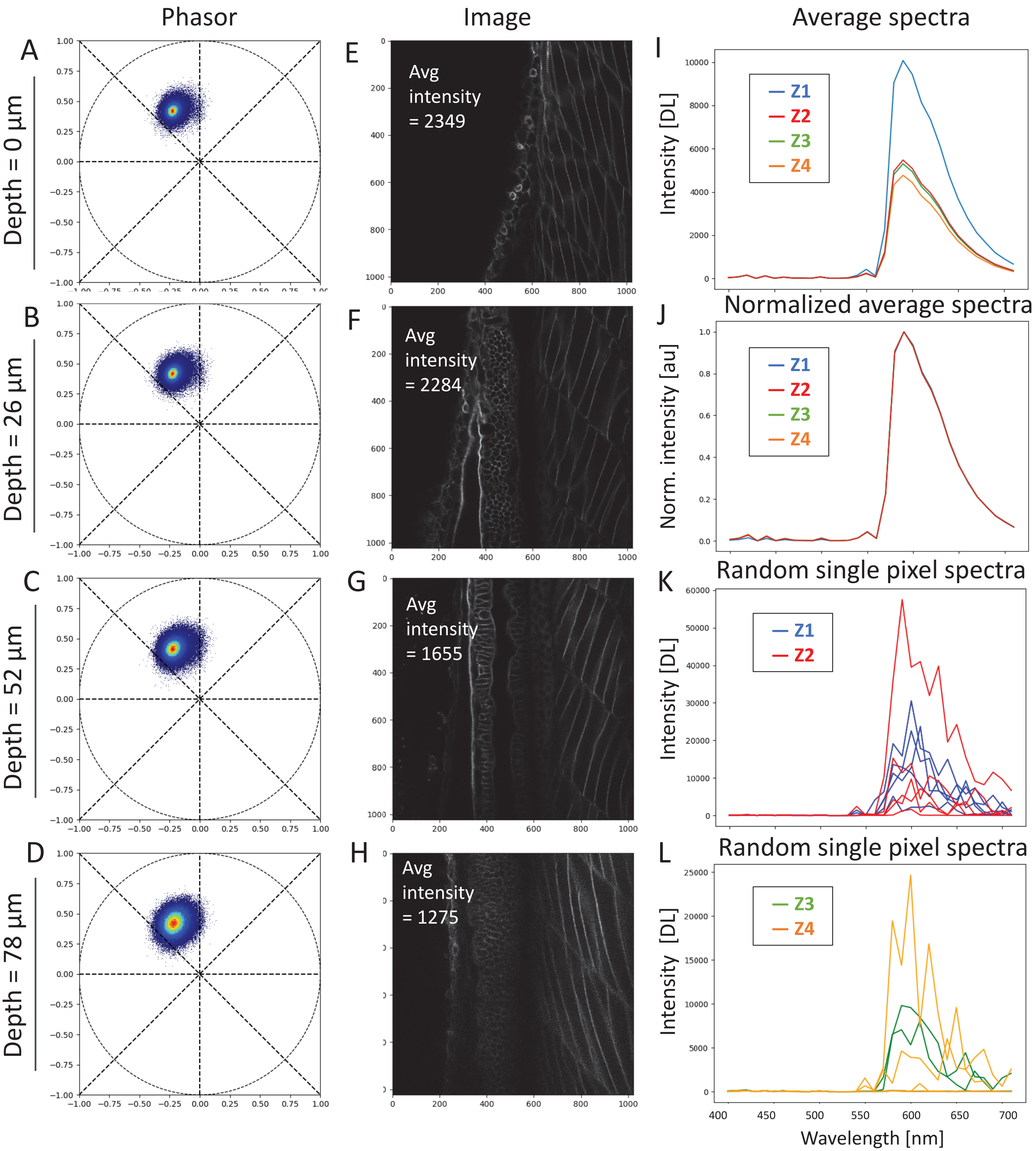
Phasor
Image
Average spectra
A
Depth = 0 µm
B
Depth = 26 µm
C
Depth = 52 µm
D
Depth = 78 µm
E
Avg intensity = 2349
F
Avg intensity = 2284
G
Avg intensity = 1655
H
Avg intensity = 1275
I
Intensity [DL]
Z1
Z2
Z3
Z4
Normalized average spectra
J
Norm. intensity [au]
Z1
Z2
Z3
Z4
Random single pixel spectra
K
Intensity [DL]
Z1
Z2
Random single pixel spectra
L
Intensity [DL]
Z3
Z4
Wavelength [nm]

Supplementary Figure 23. Phasor analysis on signal distortion in deep tissue
Images at different Z-positions of a 3D (x,y,z) dataset of 19 hpf *Tg(ubiq:lyn-tdTomato)* zebrafish acquired from 0 um to 80 um (relative to the dataset) depth displaying at every 13 z-slices. **(A-D)** Phasors calculated from the single slices at 0 µm, 26 µm, 52 µm, and 78 µm depth. **(E-H)** Corresponding average intensity images (across the 32 spectral channels) for each z-slice show an expected decrease in fluorescence intensity with depth. **(I)** Average spectra of the pixels linked to the phasor position bin for the tdTomato fluorescent signature for each of the four presented z-slices (0, 26, 52, 78 µm) show a decrease in the spectral area without change in spectral shape as shown by **(J)** normalizing the average spectra shown in **I** to each spectrum's maximum value. This demonstrates the spectral shape does not change across different depths (z-planes), while the overall intensity decreases. **(K)** Randomly selected spectra from the raw spectral image at 0 µm (blue) and 26µm (red), five spectra for each image and similarly **(L)** for 52 µm (green) and 78µm (yellow). Two yellow and three green spectra are not clearly visible because of low signal intensity.

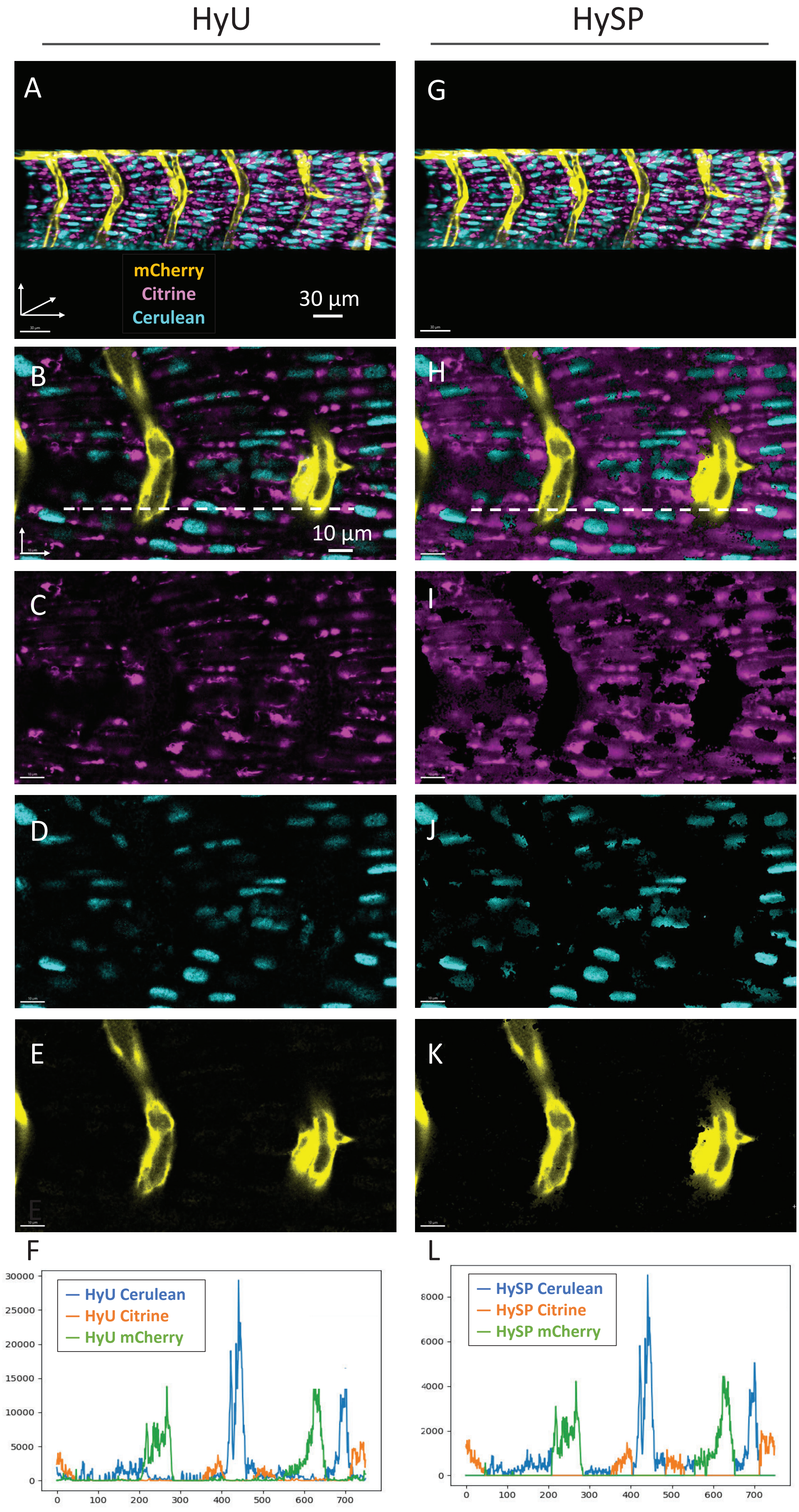
HyU
HySP
A
G
mCherry
Citrine
Cerulean
30 µm
B
H
10 µm
C
I
D
J
E
K
F
L
HyU Cerulean
HyU Citrine
HyU mCherry
HySP Cerulean
HySP Citrine
HySP mCherry
0
5000
10000
15000
20000
25000
30000
2000
4000
6000
8000
100
200
300
400
500
600
700

Supplementary Figure 24. Comparison of HyU vs HySP results from a spectrally overlapping and spatially disperse sample

Results are presented for unmixing using HyU and Hyperspectral Phasors (HySP) on a spectrally overlapping spatially disperse dataset collected from a tri-labeled transgenic zebrafish embryo obtained by injecting mRNA-encoding H2B–cerulean (cyan) in double transgenic embryos *Gt(desm-citrine)* $^{ct122a/+}$*;Tg(kdrl:eGFP)* (magenta and yellow, respectively) **(A-F)** HyU unmixing results and **(G-L)** HySP unmixing results renderings for the dataset. Line profiles of **(F)** HySP **(L)** HyU analysis results (B, H dashed line) show the similarity in signal between the two methods for all channels within a non-overlapping sample. **(A,F)** Volumetric images show a similarity between the HyU and HySP results. This is further demonstrated for the results in a **(B,H)** single z-slice, for just the **(C,I)** Citrine channel, the **(D,J)** Cerulean channel, and the **(E,K)** mCherry channel. **(F,L)** Line profiles for the lines shown in **B** and **H**, respectively, also demonstrate the similar results of HyU and HySP for spatially non-overlapping samples.

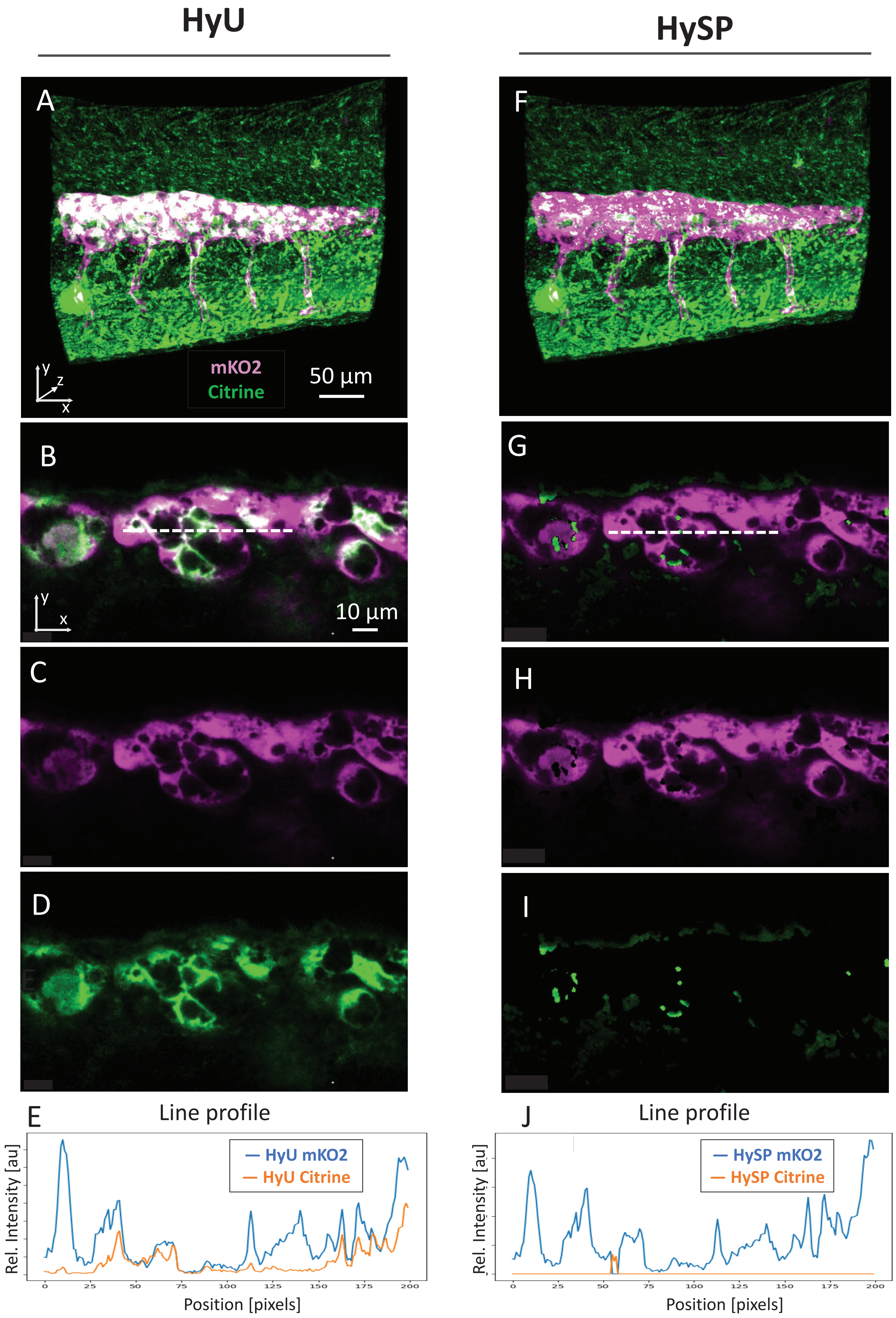
HyU
HySP
A
mKO2
Citrine
50 µm
y
z
x
F
B
10 µm
y
x
G
C
H
D
I
E
Line profile
HyU mKO2
HyU Citrine
Rel. Intensity [au]
Position [pixels]
J
Line profile
HySP mKO2
HySP Citrine
Rel. Intensity [au]
Position [pixels]

Supplementary Figure 25. Comparison of HyU vs HySP results from a spectrally overlapping and spatially non-disperse sample

Results for unmixing using HyU and HySP on a spectrally overlapping and spatially non-disperse dataset collected from a 5 dpf dual-labeled transgenic zebrafish embryo: *Gt(cltca-citrine);Tg(fli1:mKO2)*, presenting frequent combinations of signals in pixels across the dataset. **(A-E)** HyU unmixing results and **(F-K)** HySP unmixing results for the dataset. **(A,F)** Volumetric images show the expected signal overlaps between channels for the HyU result and a more distinct separation in the HySP result. This is further demonstrated for the results in a **(B,G)** single z-slice, for just the **(C,H)** mKO2 channel, and the **(D,I)** Citrine channel. **(E,J)** Line profiles for the lines shown in **B** and **G**, respectively, demonstrate the fractional nature of HyU results compared to the winner-takes-all analysis of HySP.

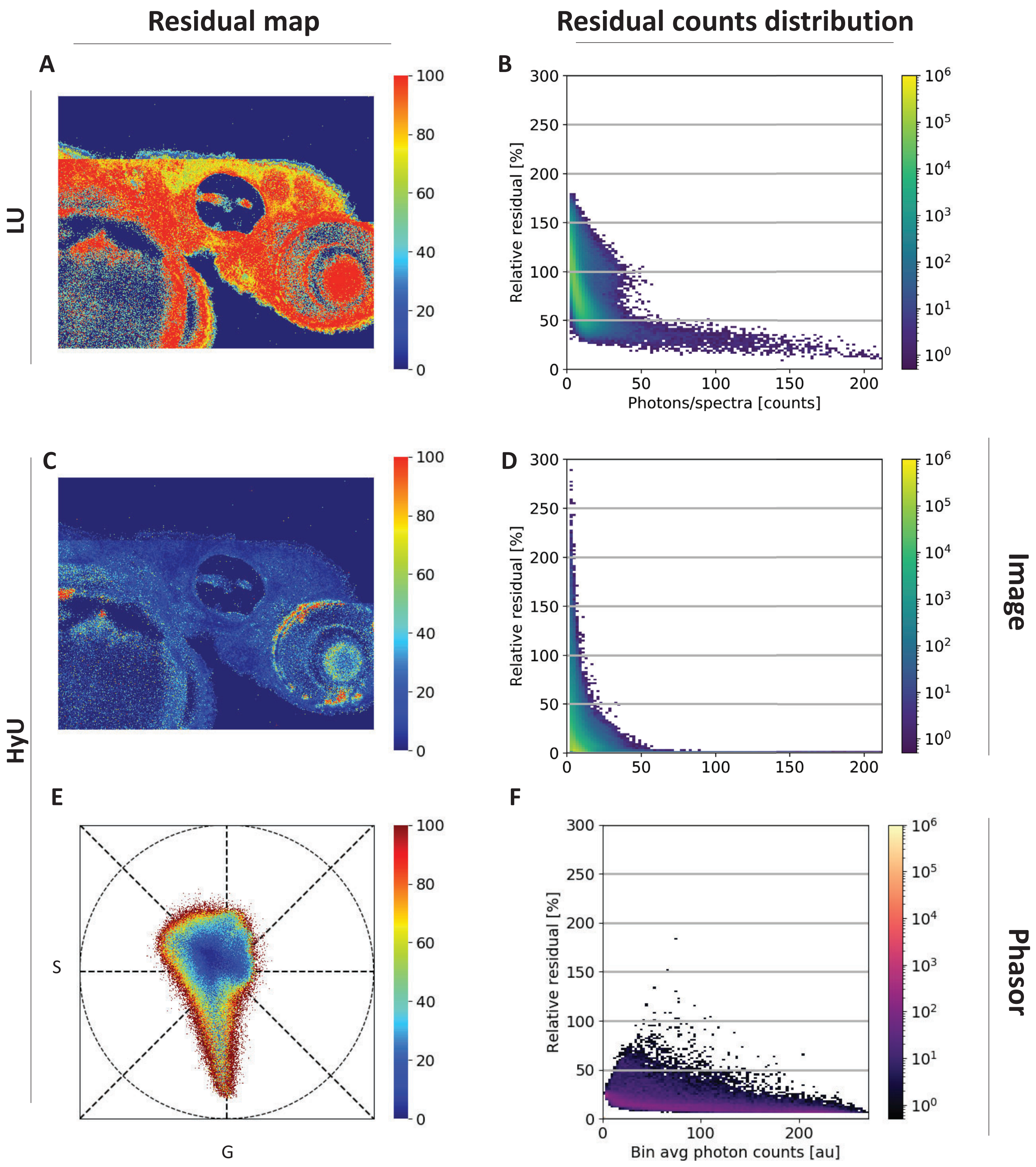

Residual map
Residual counts distribution
A
B
C
D
E
F
LU
HyU
Image
Phasor
Relative residual [%]
Photons/spectra [counts]
Bin avg photon counts [au]
S
G

Supplementary Figure 26. Residual analysis of intrinsic fluorescent signals of HyU and LU shows robust results for HyU unmixing

Residual analysis was performed on a hyperspectral fluorescent data of a 3dpf quadra-transgenic zebrafish *Gt(cltca-citrine);Tg(ubq:lyn-tdTomato;ubiq:Lifeact-mRuby;fli1:mKO2)* whose unmixing results were reported in Figure 5. Residual analysis results are shown for **(A-B)** LU and **(C-F)** HyU, respectively. Residual Image maps (Methods) of the z-averaged dataset for LU and HyU in **A** and **C**, respectively, show lower residual values for HyU, suggesting improved quality of unmixing. Residual distribution relative to the original intensity in each pixel as a function of estimated photon counts per spectrum are presented for LU and HyU in **B** and **D**, respectively, showing a distribution with lower Relative residual for HyU. **(E)** Residual Phasor Map (Methods) presents higher residual values in the regions corresponding to the edge of the phasor cluster. **(F)** Residual Phasor Histogram for HyU shows distribution of residuals in the broad dynamic range of photons for experimental data.

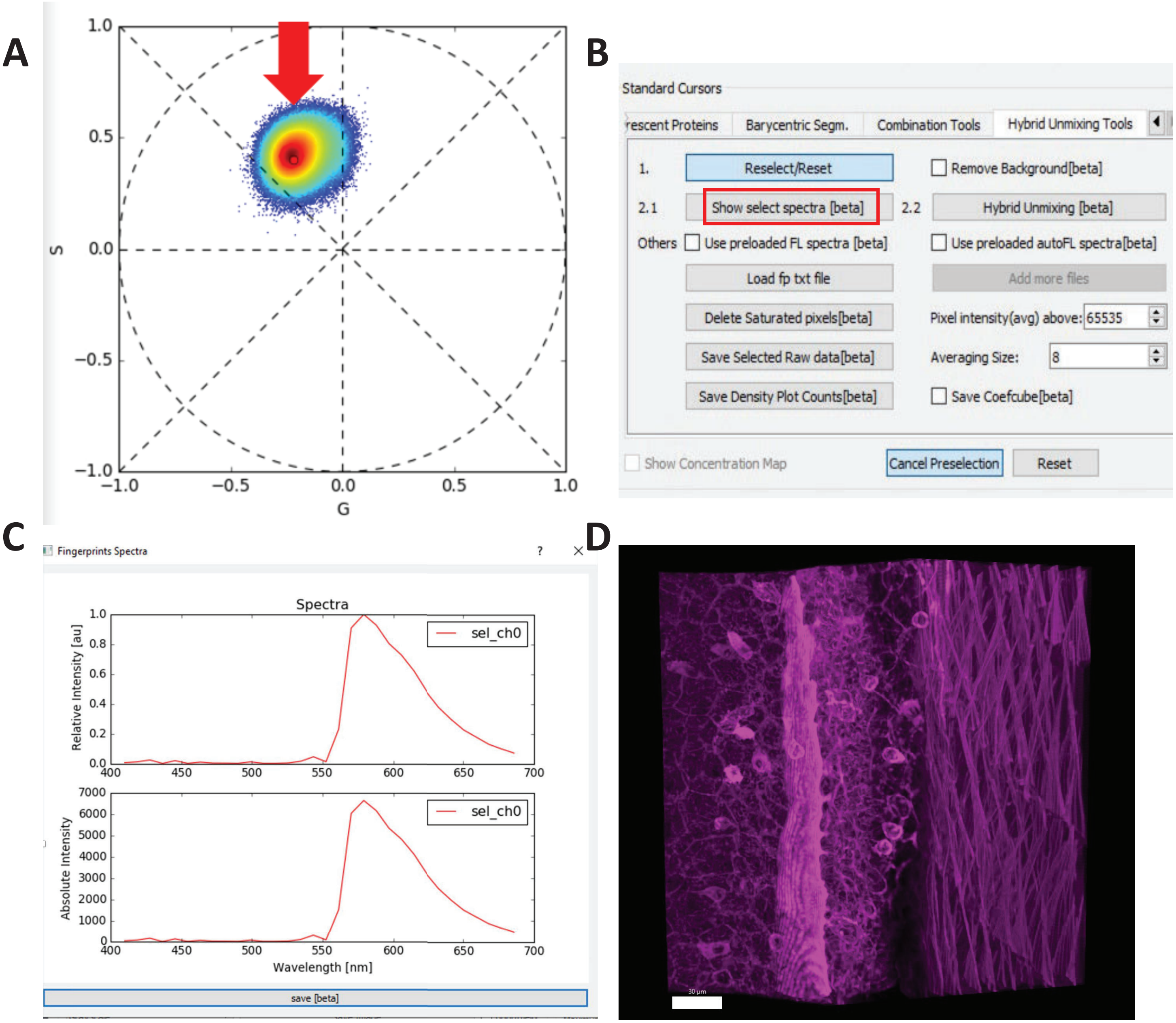
A
1.0
0.5
0.0
−0.5
−1.0
S
−1.0
−0.5
0.0
0.5
1.0
G
B
Standard Cursors
rescent Proteins
Barycentric Segm.
Combination Tools
Hybrid Unmixing Tools
1.
Reselect/Reset
Remove Background[beta]
2.1
Show select spectra [beta]
2.2
Hybrid Unmixing [beta]
Others
Use preloaded FL spectra [beta]
Use preloaded autoFL spectra[beta]
Load fp txt file
Add more files
Delete Saturated pixels[beta]
Pixel intensity(avg) above: 65535
Save Selected Raw data[beta]
Averaging Size: 8
Save Density Plot Counts[beta]
Save Coefcube[beta]
Show Concentration Map
Cancel Preselection
Reset
C
Fingerprints Spectra
Spectra
Relative Intensity [au]
1.0
0.8
0.6
0.4
0.2
0.0
sel_ch0
400
450
500
550
600
650
700
Absolute Intensity
7000
6000
5000
4000
3000
2000
1000
0
sel_ch0
Wavelength [nm]
save [beta]
D

Supplementary Figure 27. Endmember Spectrum selection process

(A) Phasor map shows the spectral distribution of the data for a single fluorescent labelled sample, in this case an 18 hpf transgenic *Tg(ubiq:lyn-tdTomato)* zebrafish. **(B)** The average spectrum corresponding to a phasor bin selection (red point in A) can be visualized using our software plotting **(C)** the corresponding average spectrum with relative (top) and absolute (bottom) intensity. The save button allows exporting spectral data as a txt file that can be re-loaded for unmixing other data. **(D)** Unmixing result. More step-by-step information is available in the README file associated with the software in this publication.

A

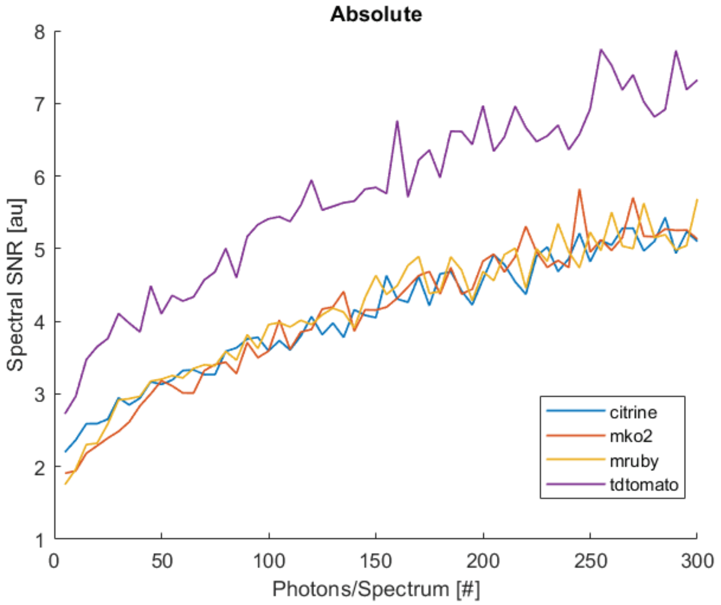
Absolute
8
7
6
5
4
3
2
1
0
50
100
150
200
250
300
Spectral SNR [au]
Photons/Spectrum [#]
citrine
mko2
mruby
tdtomato

B

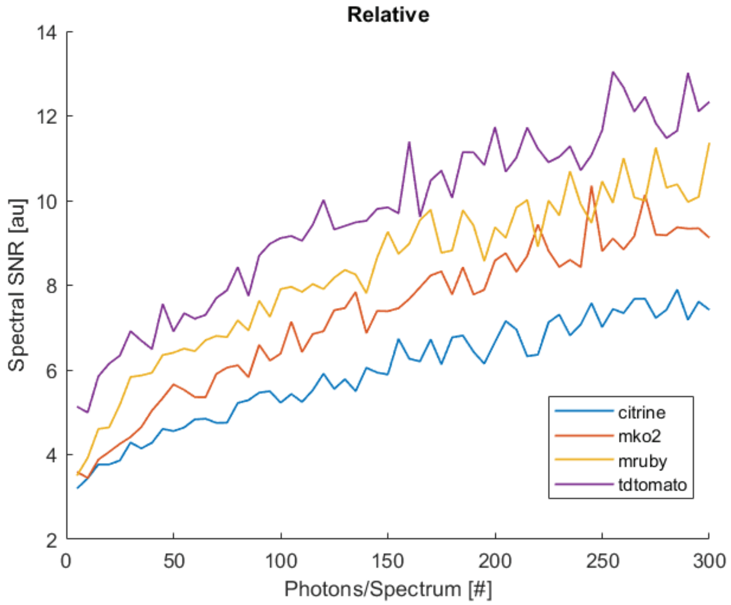
Relative
14
12
10
8
6
4
2
0
50
100
150
200
250
300
Spectral SNR [au]
Photons/Spectrum [#]
citrine
mko2
mruby
tdtomato

Supplementary Figure 28. Relationship between Spectral SNR and Photon/Spectrum
The direct relationship between SNR and photons per spectrum is shown here using the calculation of Spectral SNR for varying levels of photons per spectrum. The spectral SNR has a general trend of increased values with increasing photons per spectrum, but it is not a truly monotonic function. This non-monotonicity demonstrates the limitations of SNR when analyzing spectral images. **(A)** Absolute Spectral SNR and **(B)** Relative Spectral SNR follow the same trends of higher values with increasing photons per spectrum. However, the Relative Spectral SNR better differentiates the effects of the differing spectral shapes on the SNR. Citrine, mKO2, mRuby, and tdTomato each have easily distinguished values for the slope of the regression in ascending order. tdTomato has a spectral shape which provides the best SNR while Citrine provides the worst SNR, even with the same number of photons per spectrum.

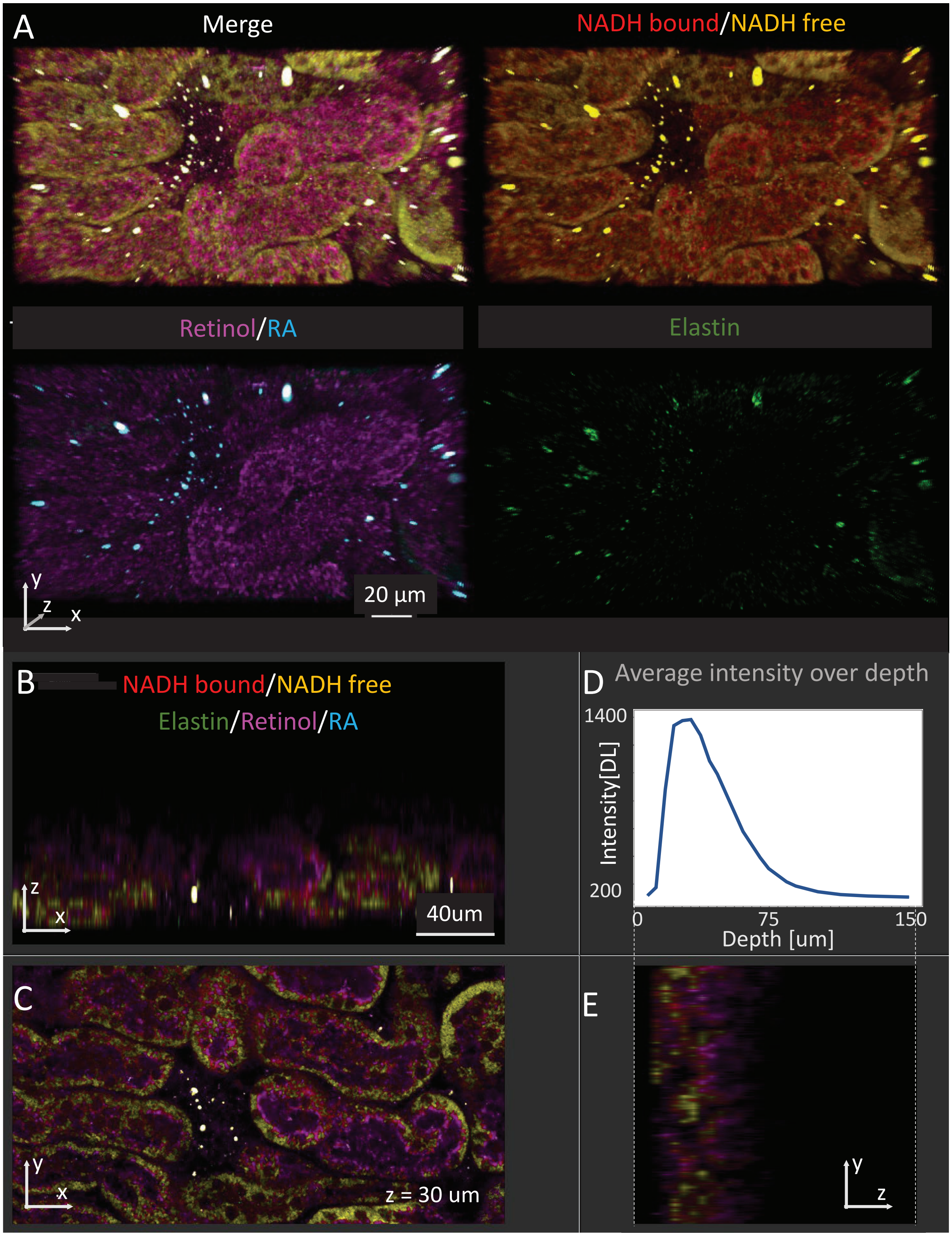
A
Merge
NADH bound/NADH free
Retinol/RA
Elastin
y
z
x
20 µm
B
NADH bound/NADH free
Elastin/Retinol/RA
z
x
40um
D
Average intensity over depth
1400
200
Intensity[DL]
0
75
150
Depth [um]
C
y
x
z = 30 um
E
y
z

Supplementary Figure 29. Intrinsic fluorescent signatures in fresh mouse tissue
Intrinsic fluorescent signatures in fresh kidney tissue of a 7 months Balb-c mouse imaged with 2-photon excitation at 740nm in a 150 µm deep volume. Despite the increasing scattering effect of this mammal tissue with increasing depth, HyU can perform unmixing of intrinsic fluorescent signals. **(A)** Volumetric rendering of the unmixing results of five intrinsic fluorescent signatures shows results consistent with literature, as visible in the **(B-E)** orthogonal views of **(C)** an unmixed (x,y) cross-section of the volume at 30 µm depth in the sample and its corresponding **(B)** (x,z) and **(E)** (y,z) projections. **(D)** Averaged autofluorescent signals for each acquired spectral (x,y) section over the 150 µm depth of the volume show a sharp decrease of intensities after 75 µm depth as visible in **E**, the corresponding (y,z) projection**.**

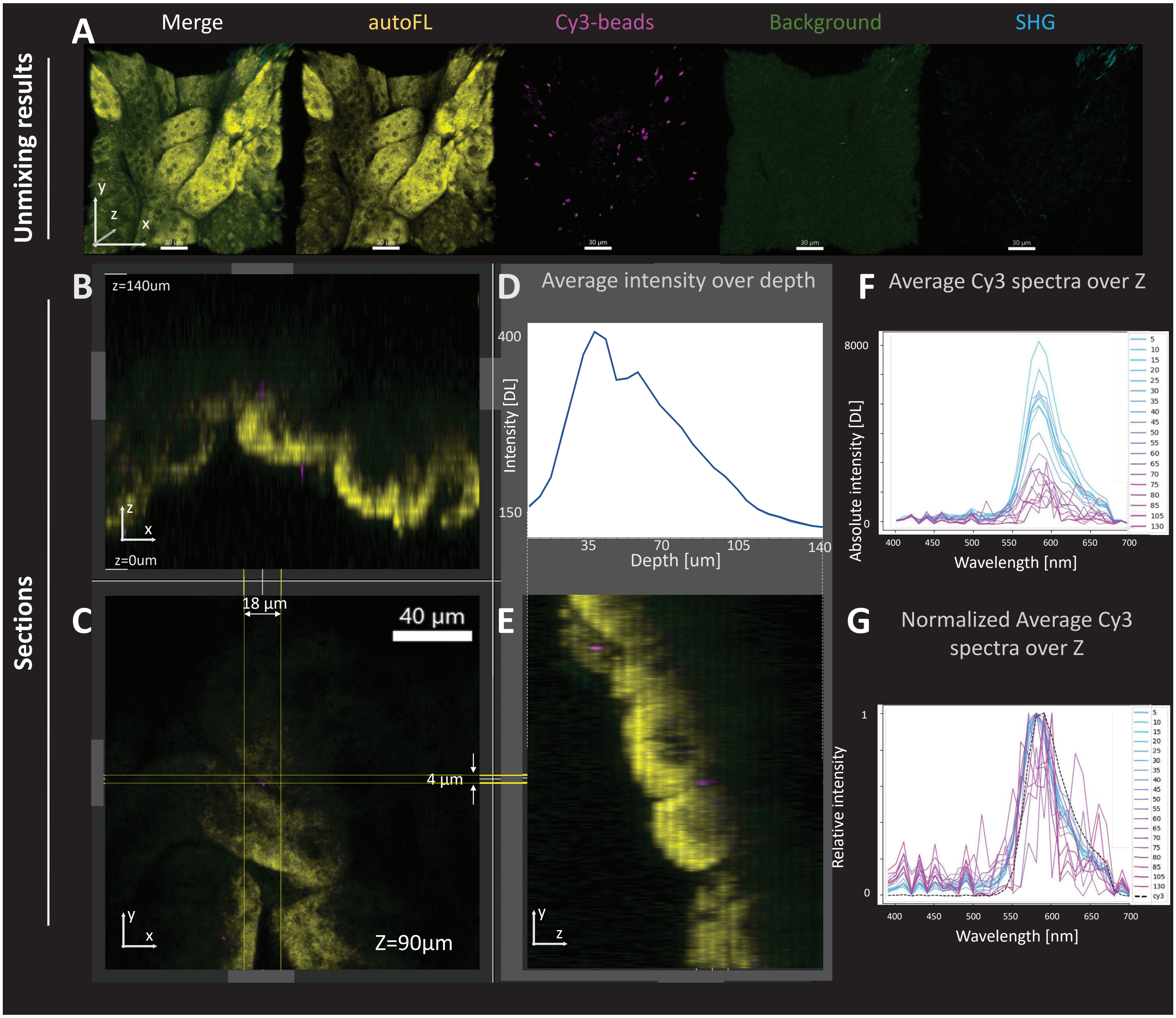
A
Merge
autoFL
Cy3-beads
Background
SHG
Unmixing results
Sections
B
z=140um
z=0um
C
18 µm
40 µm
4 µm
Z=90µm
D
Average intensity over depth
Intensity [DL]
Depth [um]
E
F
Average Cy3 spectra over Z
Absolute intensity [DL]
Wavelength [nm]
G
Normalized Average Cy3 spectra over Z
Relative intensity
Wavelength [nm]

Supplementary Figure 30. Extrinsic fluorescent signatures in fixed mouse tissue

We evaluate the performance of HyU in imaging fluorescent signals in a highly scattering fixed kidney tissue of a 7-month-old Balb-c mouse with embedded Cy3 fluorescent beads (Methods) imaged with 2-photon 850nm excitation up to 150 µm deep. **(A)** Volumetric rendering of the unmixing results of the signals from fixative autofluorescence (autoFL), Cy3 beads, background, and Second Harmonic Generation (SHG). **(B-E)** Orthogonal views of the same volume for (**C**) single (x,y) plane of the volume at a depth of 90 µm with cross-sections (yellow hairlines in C) (**B**) (z,x) of 18 µm and (**E**) (y,z) of 4 µm respectively showing sections of the unmixed volume containing Cy3 beads at different depths up to 140 µm. (**D**) Average intensity value for each acquired (x,y) spectral image slice as a function of depth reveals considerable loss of fluorescent signal deeper than 110 µm. (**F**) Average spectra for each z-plane containing pixels with Cy3 beads signal plotted with absolute intensity (Digital Levels, DL) show decreasing intensity with depth as visible by the area under spectrum. (**G**) The same averaged spectra are normalized and plotted with Relative Intensity to show the consistency in the spectral shape as a function of depth in reference to Cy3 beads in solution (dashed line).

Table S1 :

	Label	Zebrafish Stage	Imaged volume [pixels]	Imaged volume [pixels]	Imaged volume [pixels]	Lateral pixel (x,y,res.) [µm]	Axial section (z res.) [µm]	Pixel dwell time [µs]	Laser Power
Fig 2E-P, S7, Mov1	*Gt(cltca-Citrine); Tg(ubiq:lyn-tdTomato; ubiq:Lifeact-mRuby; fli1:mKO2)*	10 dpf	1024	1024	17	0.346	3.00	3.1	561 nm: 0.18%<br>488nm: 5%
Fig 3A-F, S1, S8	*Gt(cltca-Citrine); Tg(ubiq:lyn-tdTomato; ubiq:Lifeact-mRuby; fli1:mKO2)*	2 dpf	1024	512	31	0.415	5.00	3.1	561 nm: 0.6%<br>488 nm: 5.5%
Fig 4, Mov2	*Gt(cltca-Citrine); Tg(kdrl:mCherry; fli1:mKO2)*	5 dpf	512	1024	26	0.461	3.00	2.5	561 nm: 2%<br>488nm: 4%
Fig 5 A, S2	*Gt(cltca-Citrine); Tg(ubiq:lyn-tdTomato; ubiq:Lifeact-mRuby)*	3 dpf	2304	512	27	1.38	5.00	5.0	561 nm: 0.4%<br>488 nm: 2.8%
Fig 5 B-D	*Gt(cltca-Citrine); Tg(ubiq:lyn-tdTomato; ubiq:Lifeact-mRuby; fli1:mKO2)* Plus strong autofl	3 dpf	2560	2048	27	0.259	4.00	3.1	561 nm: 0.18%<br>488 nm: 5%
Fig5 E-G, S26									740 nm: 4%
Fig 6, S9,	*Gt(cltca-Citrine); Tg(ubiq:lyn-tdTomato;*	3 dpf	768	512	17	0.923	4.00	6.3	561 nm: 0.4%<br>488 nm: 5%

S10, S11, S15 Mov3	*ubiq:Lifeact-mRuby; fli1:mKO2)* Plus strong autofl								740 nm: 3%
S12	casper	36 hpf	6144	1536	25	0.461	6.00	3.1	740 nm: 3%
S22	Wildtype	22 hfp	512	512	23	0.078	2	3.1	740 nm: 2.8%
S23, S27	*Tg(ubiq:lyn-tdTomato)*	18 hpf	1024	1024	40	0.231	2	3.1	561 nm: 0.04%
S24C	*Gt(desm-citrine) ct122a/+;Tg(kdrl:eGFP)*	3 dfp	2560	2048	29	0.277	5	3.1	561 nm: 0.1%
S25	*Gt(cltca-Citrine); Tg(fli1:mKO2)*	5 dfp	1024	1024	30	0,346	2	3.1	561 nm: 0.4%<br>488 nm: 4.5%
	**Label**	**Mouse Stage**	**Imaged volume** [pixels]	**Imaged volume** [pixels]	**Imaged volume** [pixels]	**Lateral pixel (x,y,res.)** [µm]	**Axial section (z res.)** [µm]	**Pixel dwell time** [µs]	**Laser Power**
S29	Balb-c mice	7 months	973	512	31	0.277	5	6.27	740 nm: 3.5%
S30	Balb-c mice	7 months	512	512	28	0.346	5	6.27	850 nm: 4%

*hpf = hours post fertilization
*dpf = days post fertilization

Supplementary Note 1: Identification of spectra and new components with HyU

Identification of independent spectral components has been an adversity for unmixing hyperspectral data. First, the collected spectra may be distorted by reduced SNR. Secondly, excitation of intrinsic signals causes uncertainty of biological sample. Favorably, HyU simplifies this process by adapting Phasor approach and achieving semi- or full- automation process for spectra identification and selection. In HyU, spectra can be loaded from an existing library, virtually automating the analysis process. Pre-identified cursors are generated from common fluorophores such as mKO2, tdTomato, mRuby, Citrine. In our experience, obtaining fluorescence spectra from experimental samples has some advantages compared to utilizing spectra from an existing library, as they account for a multitude of experimental and instrumental settings. Imaging settings such as different types of lenses or optical filters (Sup. Figure 4, C and D) together with factors within the microenvironment of samples, such as pH or temperature have the potential to alter the fluorescence spectral emissions[3]. In the presence of unexpected fluorescent signals, spectra can also be selected and visualized directly from the phasor. Phasors facilitate the identification of unexpected independent components and their distinction from the multiple system noises. A noise-free spectrum will appear as a single point on the phasor plot, while a spectrum affected by instrument and electronic noises will mainly appear as a gaussian distribution, centered on the original spectral signal[1]. Conversely, a randomized noise across the multiple spectral channels will not produce a clustered aggregate of spectra on the phasor. A constant spectral noise, with a distinct spectrum (e.g. a constant light leakage into the system), would produce a distinct phasor cluster and could be selected for unmixing. The phasor plot representation is a 2D-histogram and provides insights into the frequency of occurrence for these signals. These unexpected independent components in samples often appear as “tails” on the phasor distributions (Sup. Figure 11, C). In our HyU graphical interface, clicking on the phasor visualizes the spectra within a small area (9x9 bins by default, with size adjustable from the interface) of the phasor histogram (Figure 1 D). In the example in Sup. Figure 9-11, we identify 5 distinct endmembers on the Phasor (Sup. Figure 10, C), visualize their spectra identifying Citrine, mRuby, Td-Tomato, mKO2, and one strong autofluorescence signature. The use of Residual Phasor Map (Sup. Figure 11, B) allows for identification of areas in the phasor with high amount of residuals, likely corresponding to a missing endmember in the unmixing. Residual Image Maps (Sup. Figure 11, C) provide a rapid overview of residuals in the image data, for identification of location in the dataset of the missing endmember.

Supplementary Note 2: HyU in autofluorescent data

Cellular metabolism is a key regulator of cell function and plays an essential role in the development of numerous diseases. Understanding of cellular metabolic pathways is critical for the development and assessment of novel therapies and diagnostics. A number of metabolites have been reported in literature to be fluorescent and to change their spectra according to their biochemical configurations. For example, the measurement of NADH in its free and bound state is possible thanks to a shift in the emission spectra when NADH is bound to enzymes such as Lactate Dehydrogenase (LDH). Likewise, retinol and retinoic are known to have different autofluorescent spectra. A map of the phasor position for common autofluorescence from pure solutions is reported in Sup. Figure 21 B. Imaging autofluorescent data, with regard to cell metabolism, requires accounting for complex and dynamic changes of metabolic pathways which can occur in a broad range of times, from seconds to years. These autofluorescent signals are often weak in nature and do not rapidly replenish after photobleaching. In our work we utilize reduced laser power to avoid rapid autofluorescence spectral signal bleaching, as well to reduce photo-damage. Additional factors known to affect emission spectra include pH and temperature, pixel-wise concentration of the fluorophore, excitation power, developmental stage and region of the sample imaged. An example for the latter is reported in Sup. Figure 12, where signals in the sample present strong localized differences. One example of the effects of different 2-photon excitation power and different levels of pixel-wise concentration is reported in Figure 5, Figure 6. In these images, samples at similar developmental stages are imaged utilizing different pixel size (0.259 µm and 0.923 µm lateral resolutions) and laser power (4% and 3% @740nm 2-photon) resulting in laser power densities of ~ $4.7 \cdot 10^{-6}$ mW/mm^2. This different laser power causes some lower-concentration intrinsic fluorophores to not be excited, in this case mRuby is visible in Figure 5 but not in Figure 6. In both of these images, FAD is not excited in measurable quantity, whereas in Sup. Figure 22, where the laser power density is $1.4 \cdot 10^{-3}$ mW/mm^2, FAD contribution is measurable and unmixed. HyU is well posed for the analysis of intrinsically low autofluorescence owing to its ability to operate at low SNR. In Sup. Figure 12, we visualize unmixing of multiple autofluorescent signals based on spectra acquired from in vitro solutions. Sup. Figures 17, 19 present a simulated overview of the improvement of HyU over Linear Unmixing for autofluorescence data, as a function of number of labels, percentage of pixels containing mixed ratio of fluorophores, number of denoising filters applied and number of channels under different levels of Signal to Noise.

Supplementary Note 3: Reduced computational costs during unmixing

An advantage of HyU is speed. HyU provides substantial speed boosts when comparing to other pixel-based unmixing algorithms. The exception is for standard LU vs HyU owing to the highly optimized computational implementation of the functions which are utilized in standard LU. This speed boost occurs because unmixing is performed at a phasor-histogram level, where a single bin corresponds to a multitude of image pixels. For algorithms other than standard LU, HyU provides up to ~500-fold improvement in speed at comparable coding language and computing hardware, processing 2 GB in less than 100 seconds (Supplementary Figure 13).

This improvement provides a solution for open image-analysis challenges in multiplexing fluorescence. First, the increased size of HFI data, resulting from continuously higher throughput and resolution microscopes, scaled with the number of spectral channels. Second, the number of datasets, owing to experimental reproducibility and biological variability.

Supplementary Note 4: Improvements of HyU over the standard phasor analysis

Linearity of combinations is the general assumption for most of the spectral analysis algorithms in Hyperspectral Fluorescence Imaging (HFI). Each pixel is assumed to contain a linear combination of the independent spectral signatures, or endmembers, contained in the sample. This assumption requires knowledge, or identification, of the independent spectra within the sample. In standard linear unmixing algorithms, the extraction of relative amounts of spectra (ratios) is conducted on a pixel-by-pixel basis, at the expense of computational costs. Disrupted experimental signals, in the case of lower Signal to Noise Ratio (SNR) spectra, complicate the detection of spectral endmembers and reduce the accuracy of ratio determination. These standard unmixing algorithms, however, have the advantage of being unsupervised with the possibility of automating the analysis process.

The phasor approach has become a popular dimensionality reduction approach for the analysis of both fluorescence lifetime and spectral image analysis[2,3]. Phasors provide key advantages, including spectral compression, denoising, and computational reduction for both pre-processing[4] and unmixing[1,5,6] of HFI datasets. Phasor analysis overcomes the challenge of low SNR data analysis that limits standard unmixing algorithms, providing a multiplexing solution to a need. The phasor transform is a lossy encoder that in principle carries a reduced percentage of the information compared to the original clean data[1] In the imaging of fluorescent signals, where signal to noise often decreases to lower digits, the encoding loss is less relevant compared to the noise of the fluorescent signals. This fundamental advantage of increasing SNR in noisy data has made the phasor method a valuable tool for fluorescence microscopy, both for Lifetime and Spectral Fluorescence Microscopy. This point is reported by multiple groups using phasors[1,6–8] and, more recently, nicely described in the work of Scipioni et al[9]. Standard Phasor analysis[10–12] is fully supervised and requires a manual selection of regions or points on a graphical representation of the transformed spectra, called the phasor plot. Each selection of a region in the phasor plot associates pixels containing similar spectra to the same fluorophore, forming an output channel that contains wavelength integral of intensities with unitary ratiometric value. This "winner takes all" approach is suitable when fluorophores for each single excitation light are spectrally overlapping and spatially disperse (Sup. Figure 24), but requires separate acquisition of different excitation wavelengths for demultiplexing spatially and spectrally overlapping fluorophores (Sup. Figure 25).

HyU uses the phasor transform to group pixels with similar spectral shape within each phasor histogram bin. This approach maintains the advantage of compressing, denoising and simplifying identification of clean endmember fluorescent spectra. However, HyU improves on the robustness of the analysis. The denoised signals are maintained in a hybrid phasor and wavelength domain, and therefore can be unmixed with a multitude of standard unmixing algorithms (Sup. Figure 13), such as Linear Unmixing or Fully Constrained Least Squares. These standard unmixing approaches can operate without supervision and provide for each pixel the ratios for a set of spectral signals, overcoming some of the limitations of phasor, but generally do not perform well in experimental conditions with reduced and compromised signals, such as in fluorescence, and require extensive computational time for high spectral-count datasets. HyU provides wavelength-based denoised spectra that enable these standard algorithms to outperform their pixel-by-pixel typical application in quality of the results (Sup. Figures 16-19), owing to cleaner and better-defined fluorescent spectra in each phasor bin, and, generally, in speed, owing to the phasor dimensionality reduction. HyU performs well for single excitation

light when fluorophores are spectrally overlapping both when they are spatially disperse or co-localized, providing a ratio for each independent spectrum currently unmixed. Our data suggests HyU has reasonable performance for up to 8 different fluorophores per dataset, for each single excitation wavelength. In an experiment with a carefully chosen palette of labels, where octuples of fluorophores can be excited by a single wavelength, with an instrument capable of spectral acquisition with 5 standard and sufficiently spectrally separated excitation wavelengths in 5 sequential acquisitions (one for each excitation light), HyU could, in principle, unmix 40 signals. This performance however decreases with the number of channels (Sup. Figures 18, 19) showing a small deterioration at 8 channels and limitations at 4.

1. Cutrale F, Trivedi V, Trinh LA, et al. Hyperspectral phasor analysis enables multiplexed 5D in vivo imaging. *Nat Methods*. 2017;14(2):149-152. doi:10.1038/nmeth.4134
2. Shimozono S, Iimura T, Kitaguchi T, Higashijima SI, Miyawaki A. Visualization of an endogenous retinoic acid gradient across embryonic development. *Nature*. 2013;496(7445). doi:10.1038/nature12037
3. Islam MS, Honma M, Nakabayashi T, Kinjo M, Ohta N. pH dependence of the fluorescence lifetime of FAD in solution and in cells. *Int J Mol Sci*. 2013;14(1). doi:10.3390/ijms14011952
4. Shi W, Koo DES, Kitano M, et al. Pre-processing visualization of hyperspectral fluorescent data with Spectrally Encoded Enhanced Representations. *Nat Commun*. 2020;11(1):1-15. doi:10.1038/s41467-020-14486-8
5. Andrews LM, Jones MR, Digman MA, Gratton E. Spectral phasor analysis of Pyronin Y labeled RNA microenvironments in living cells. *Biomed Opt Express*. 2013;4(1). doi:10.1364/boe.4.000171
6. Fereidouni F, Bader AN, Gerritsen HC. Spectral phasor analysis allows rapid and reliable unmixing of fluorescence microscopy spectral images. *Opt Express*. 2012;20(12). doi:10.1364/oe.20.012729
7. Stringari C, Cinquin A, Cinquin O, Digman MA, Donovan PJ, Gratton E. Phasor approach to fluorescence lifetime microscopy distinguishes different metabolic states of germ cells in a live tissue. *Proc Natl Acad Sci U S A*. 2011;108(33). doi:10.1073/pnas.1108161108
8. Lanzanò L, Coto Hernández I, Castello M, Gratton E, Diaspro A, Vicidomini G. Encoding and decoding spatio-temporal information for super-resolution microscopy. *Nat Commun*. 2015;6. doi:10.1038/ncomms7701
9. Scipioni L, Rossetta A, Tedeschi G, Gratton E. Phasor S-FLIM: a new paradigm for fast and robust spectral fluorescence lifetime imaging. *Nat Methods*. 2021;18(5). doi:10.1038/s41592-021-01108-4
10. Malacrida L, Ranjit S, Jameson DM, Gratton E. The Phasor Plot: A Universal Circle to Advance Fluorescence Lifetime Analysis and Interpretation. *Annu Rev Biophys*. 2021;50. doi:10.1146/annurev-biophys-062920-063631
11. Ranjit S, Malacrida L, Jameson DM, Gratton E. Fit-free analysis of fluorescence lifetime imaging data using the phasor approach. *Nat Protoc*. 2018;13(9):1979-2004. doi:10.1038/s41596-018-0026-5
12. Digman MA, Caiolfa VR, Zamai M, Gratton E. The phasor approach to fluorescence lifetime imaging analysis. *Biophys J*. 2008;94(2). doi:10.1529/biophysj.107.120154
