# Supplementary material for: HyU: Hybrid Unmixing for longitudinal in vivo imaging of low signal-to-noise fluorescence
Source: Nat Methods. 2023 Jan 19;20(2):248–58. doi: 10.1038/s41592-022-01751-5 (PMC9911352; doi:10.1038/s41592-022-01751-5)
Supplement: Supplementary file 2 — Reporting Summary [file 41592_2022_1751_MOESM2_ESM.pdf]

nature research

Corresponding author(s): Francesco Cutrale

Last updated by author(s): Nov 3, 2022

Reporting Summary

Nature Research wishes to improve the reproducibility of the work that we publish. This form provides structure for consistency and transparency in reporting. For further information on Nature Research policies, see our Editorial Policies and the Editorial Policy Checklist.

Statistics

For all statistical analyses, confirm that the following items are present in the figure legend, table legend, main text, or Methods section.

n/a	Confirmed	
☐	☒	The exact sample size (*n*) for each experimental group/condition, given as a discrete number and unit of measurement
☐	☒	A statement on whether measurements were taken from distinct samples or whether the same sample was measured repeatedly
☐	☒	The statistical test(s) used AND whether they are one- or two-sided<br>*Only common tests should be described solely by name; describe more complex techniques in the Methods section.*
☒	☐	A description of all covariates tested
☒	☐	A description of any assumptions or corrections, such as tests of normality and adjustment for multiple comparisons
☐	☒	A full description of the statistical parameters including central tendency (e.g. means) or other basic estimates (e.g. regression coefficient) AND variation (e.g. standard deviation) or associated estimates of uncertainty (e.g. confidence intervals)
☐	☒	For null hypothesis testing, the test statistic (e.g. *F*, *t*, *r*) with confidence intervals, effect sizes, degrees of freedom and *P* value noted<br>*Give P values as exact values whenever suitable.*
☒	☐	For Bayesian analysis, information on the choice of priors and Markov chain Monte Carlo settings
☒	☐	For hierarchical and complex designs, identification of the appropriate level for tests and full reporting of outcomes
☒	☐	Estimates of effect sizes (e.g. Cohen's *d*, Pearson's *r*), indicating how they were calculated

Our web collection on statistics for biologists contains articles on many of the points above.

Software and code

Policy information about availability of computer code

Data collection	Commercial software Zen Black 2.3 SP1 FP3, Carl Zeiss, for controlling Zeiss 780 Microscope.
Data analysis	Data was preprocessed utilizing custom python algorithms now integrated in HySP (http://bioimaging.usc.edu/software.html#HySP), data was rendered using commercial software Imaris 9.5-9.7. 3D registration was done using the Correct 3D Drift plugin in FIJI (Java 8 Life-Line version). Simulations were made using custom Python code, starting from real data.<br>All the relevant code is available from the corresponding author upon reasonable request. Software and instructions can be downloaded from http://bioimaging.usc.edu/software.html.

For manuscripts utilizing custom algorithms or software that are central to the research but not yet described in published literature, software must be made available to editors and reviewers. We strongly encourage code deposition in a community repository (e.g. GitHub). See the Nature Research guidelines for submitting code & software for further information.

Data

Policy information about availability of data

All manuscripts must include a data availability statement. This statement should provide the following information, where applicable:

- Accession codes, unique identifiers, or web links for publicly available datasets
- A list of figures that have associated raw data
- A description of any restrictions on data availability

Data and materials availability: All the relevant data are available from the corresponding author upon reasonable request. Datasets for Figs. 1–6 and simulations are available for download at http://bioimaging.usc.edu/software.html#sampledatasets in the samples section.

Field-specific reporting

Please select the one below that is the best fit for your research. If you are not sure, read the appropriate sections before making your selection.

☒ Life sciences ☐ Behavioural & social sciences ☐ Ecological, evolutionary & environmental sciences

For a reference copy of the document with all sections, see nature.com/documents/nr-reporting-summary-flat.pdf

Life sciences study design

All studies must disclose on these points even when the disclosure is negative.

Sample size	No sample size was predetermined. The work here presented describes an analysis method which is run against different fluorescent samples including autofluorescence and multiple-fluorescent labels. Samples were acquired over multiple experimental sessions until a clear performance trend was evident. Performance was then validated across multiple scenarios with simulated data.
Data exclusions	No data was excluded from analysis
Replication	Reproducibility was verified across at least 5 different samples in different days. Datasets are available for download at http://bioimaging.usc.edu/software.html#sampledatasets in the samples section while software can be downloaded from http://bioimaging.usc.edu/software.html#HySP.
Randomization	Non-pertinent, no clinical trial involved. Sample order was not considered in this work as this is an analysis method which runs on one sample per time. Order does not affect how the method works.
Blinding	Blinding was not relevant to this study as this method returns multiple images as output which require no interpretation from the user beside the capability of distinguishing colors on a screen.

Reporting for specific materials, systems and methods

We require information from authors about some types of materials, experimental systems and methods used in many studies. Here, indicate whether each material, system or method listed is relevant to your study. If you are not sure if a list item applies to your research, read the appropriate section before selecting a response.

Materials & experimental systems

n/a	Involved in the study	
☒	☐	Antibodies
☒	☐	Eukaryotic cell lines
☒	☐	Palaeontology and archaeology
☐	☒	Animals and other organisms
☒	☐	Human research participants
☒	☐	Clinical data
☒	☐	Dual use research of concern

Methods

n/a	Involved in the study	
☒	☐	ChIP-seq
☒	☐	Flow cytometry
☒	☐	MRI-based neuroimaging

Animals and other organisms

Policy information about studies involving animals; ARRIVE guidelines recommended for reporting animal research

Laboratory animals	18 to 72 hours-post-fertilization zebrafish embryos:<br>1. Gt(cltca-Citrine); Tg(ubiq:lyn-tdTomato; ubiq:Lifeact-mRuby; fli1:mKO2)<br>2. Gt(cltca-Citrine); Tg(kdrl:mCherry;fli1:mKO2)<br>3. Gt(cltca-Citrine); Tg(ubiq:lyn-tdTomato; ubiq:Lifeact-mRuby)<br>4. mpv17a9/a9;mitfaw2/w2 (casper) /csf1rj4e1/j4e1 (panther)<br>Detailed reporting of lines is in "Zebrafish lines" and Table 1 Supplementary Materials. All zebrafish lines are available from the authors.<br><br>7 month old female Balb-C mice Details are reported in "Mouse sample preparation" section and Table 1 in Supplementary Material.
Wild animals	This study did not involve wild animals
Field-collected samples	This study did not involve field-collected samples
Ethics oversight	For Zebrafish lines: IACUC of University of Southern California (permit number: 12007); For Mouse lines: IACUC of USC (permit number: 21311)

Note that full information on the approval of the study protocol must also be provided in the manuscript.
