# Supplementary material for: HyU: Hybrid Unmixing for longitudinal in vivo imaging of low signal-to-noise fluorescence
Source: Nat Methods. 2023 Jan 19;20(2):248–58. doi: 10.1038/s41592-022-01751-5 (PMC9911352; doi:10.1038/s41592-022-01751-5)
Supplement: Supplementary file 4 — Editorial Assessment Report [file 41592_2022_1751_MOESM4_ESM.pdf]

Contents of this report

1. Manuscript details: overview of your manuscript and the editorial team.
2. Review synthesis: summary of the reviewer reports provided by the editors.
3. Editorial recommendation: personalized evaluation and recommendation from all 3 journals.
4. Annotated reviewer comments: the referee reports with comments from the editors.
5. Open research evaluation: advice for adhering to best reproducibility practices.

About the editorial process

Because you selected the **Nature Portfolio Guided Open Access** option, your manuscript was assessed for suitability in three of our titles publishing high-quality work across the spectrum of methods research: ***Nature Methods*, *Nature Communications*, and *Communications Biology***. More information about Guided Open Access can be found here.

Collaborative editorial assessment

Your editorial team discussed the manuscript to determine its suitability for the Nature Portfolio Guided OA pilot. Our assessment of your manuscript takes into account several factors, including whether the work meets the **technical standard** of the Nature Portfolio and whether the findings are of **immediate significance** to the readership of at least one of the participating journals in the Nature Portfolio Guided Open Access methods cluster.

Peer review

Experts were asked to evaluate the following aspects of your manuscript:

- **Novelty** in comparison to prior publications;
- **Likely audience** of researchers in terms of broad fields of study and size;
- **Potential impact** of the study on the immediate or wider research field;
- **Evidence** for the claims and whether additional experiments or analyses could feasibly strengthen the evidence;
- **Methodological detail** and whether the manuscript is reproducible as written;
- Appropriateness of the **literature review**.

Editorial evaluation of reviews

Your editorial team discussed the potential suitability of your manuscript for each of the participating journals. They then discussed the revisions necessary in order for the work to be published, keeping each journal's specific editorial criteria in mind.

Journals in the Nature portfolio will support authors wishing to transfer their reviews and (where reviewers agree) the reviewers' identities to journals outside of Springer Nature.
If you have any questions about review portability, please contact our editorial office at guidedoa@nature.com.

Manuscript details

Tracking number	Submission date	Decision date	Peer review type
GUIDEDOA-21-00318	Nov 12, 2021	Aug 19, 2021	Single-blind

Manuscript title

HyU: Hybrid Unmixing for longitudinal in vivo imaging of low signal to noise fluorescence

Preprint: https://assets.researchsquare.com/files/rs-1073331/v1_covered.pdf?c=1642001999

Author details

Francesco Cutrale

Affiliation: University of Southern California

Editorial assessment team

**Primary editor**	**Rita Strack**<br>Home journal: ***Nature Methods***<br>ORCID: **0000-0003-1845-7116**<br>Email: **rita.strack@us.nature.com**
**Other editors consulted**	**Cara Eldridge**<br>Home journal: ***Nature Communications***<br>ORCID: **0000-0001-7001-2312**<br><br>**Anam Akhtar**<br>Home journal: ***Communications Biology***<br>ORCID: **0000-0002-8820-8468**
**About your primary editor**	Rita Strack obtained her Ph.D. in Biochemistry and Molecular Biology from the University of Chicago. While there, she worked with Benjamin Glick and Robert Keenan to engineer improved variants of the red fluorescent protein DsRed, and also studied the chemical mechanism of chromophore formation in DsRed. She continued her research as a postdoctoral fellow in Samie Jaffrey's laboratory at Weill Cornell Medical

	College, where she developed fluorescent reporters for live-cell imaging of RNA such as Spinach2. She handles imaging, microscopy and probes, along with protein and RNA biochemistry content for the journal. Rita joined Nature Methods in November 2014.

nature portfolio

GUIDED OPEN ACCESS
nature methods | nature communications | communications biology

Editorial assessment and review synthesis

**Editor's summary and assessment**	This paper describes a new strategy for separating fluorophore signals in multiplexed imaging. Their motivation is that this method is needed for low SNR imaging, which is common for extended time-lapse imaging. The method is an analytical tool suite that works with hyperspectral imaging data. Their approach, called Hybrid Unmixing (HyU), combines the power of hyperspectral phasor analysis for unmixing weak, noisy, and highly overlapping signals with the power of standard unmixing algorithms, such as linear unmixing, for removing any subjectivity in assignments. HyU offers improved unmixing, especially for low intensity images, simplified identification of spectra components, and fast processing.
**Editorial synthesis of reviewer reports**	The referees agree that that HyU will be valuable for researchers working in multiplexed imaging. However, they have concerns that will require additional experimental validation and clarifications. These concerns include (but are not limited to) concerns about the fundamental validity of using a single harmonic to do phasor transformation of more than three signals, concerns about applicability in thick/scattering tissue, and performance in situations where all reference spectra are not available. They also seek additional comparisons, especially to hyperspectral phasors, a related approach developed by Cutrale et al.. The referees were able to use the software as intended.

Editorial recommendation

Nature Methods<br><br>Major revisions with extension of the work	Nature Methods would be interested in considering a revised version that addresses all technical concerns and adds additional demonstrations. The major points for reconsideration will be:<br><br>- Results of comparison to HySp<br>- Performance on scattering tissue<br>- Adequate justification of using a single harmonic for phasor analysis of more than three components<br>- Performance when one or more spectra of imaged components is missing
Nature Communications<br><br>Major revisions	Nature Communications would also be interested in considering a revision. We request that all of the points raised by Nature Methods are addressed, but we do not absolutely require a more challenging application to assess the performance on scattering tissue.
Communications Biology<br><br>Minor revisions	Communications Biology is also interested in inviting a revision that clarifies all the points raised by the reviewers. However, we also do not ask for the point on experimental performance on scattering tissue or comparison to HySP.

Next steps

**Editorial recommendation 1:**	Our top recommendation is to revise and resubmit your manuscript to *Nature Methods.* We feel the additional experiments required are reasonable, will strengthen the manuscript, and will separate the work from the previous state-of-the-art.
**Editorial recommendation 2:**	You may also choose to revise and resubmit your manuscript to *Nature Communications.* This option might be best if the requested additional challenging demonstration is not feasible at this time.
**Editorial recommendation 3:**	Alternatively, you may choose to revise and resubmit your manuscript to Communications Biology. This option might be suitable if you are able to address most of the concerns textually.

Revision

To follow our recommendation, please upload the revised manuscript files using **the link provided in the decision letter**. Should you need assistance with our manuscript tracking system, please contact Adam Lipkin, our Nature Portfolio Guided OA support specialist, at guidedOA@nature.com.

Revision checklist

- ☐ Cover letter, stating to which journal you are submitting
- ☐ Revised manuscript
- ☐ Point-by-point response to reviews
- ☐ Updated Reporting Summary and Editorial Policy Checklist
- ☐ Supplementary materials (if applicable)

Submission elsewhere

If you choose not to follow our recommendations, you can still take the reviewer reports with you.

Option 1: Transfer to another Nature Portfolio journal
Springer Nature provides authors with the ability to transfer a manuscript within the Nature Portfolio, without the author having to upload the manuscript data again. To use this service, **please follow the transfer link provided in the decision letter.** If no link was provided, please contact guidedOA@nature.com.

> *Note that any decision to opt in to In Review at the original journal is not sent to the receiving journal on transfer. You can opt in to In Review at receiving journals that support this service by choosing to modify your manuscript on transfer.*

Option 2: Portable Peer Review option for submission to a journal outside of Nature Portfolio
If you choose to submit your revised manuscript to a journal at another publisher, we can share the reviews with another journal outside of the Nature Portfolio if requested. You will need to request that the receiving journal office contacts us at guidedOA@nature.com. We have included editorial guidance below in the reviewer reports and open research evaluation to aid in revising the manuscript for publication elsewhere.

Annotated reviewer reports

The editors have included some additional comments on specific points raised by the reviewers below, to clarify requirements for publication in the recommended journal(s). However, please note that all points should be addressed in a revision, even if an editor has not specifically commented on them.

Reviewer #1 information

**Expertise**	Mutiplexed imaging, linear unmixing, phasor analysis
**Editor's comments**	Reviewer 1 thinks the work could be suitable for Nature Methods, but is concerned that there is a fundamental flaw in the way the authors have applied phasor analysis that must be addressed by the authors.

Reviewer #1 comments

Section	Annotated Reviewer Comments
**Remarks to the Author: Overall significance**	The paper by Chiang et al titled "HyU: Hybrid Unmixing for longitudinal in vivo imaging of low signal to noise fluorescence" has a lot of details that are really attractive. This includes the possibility of multiplexing using a combination of Phasor and linear unmixing and potential for understanding the distribution pattern for multiple fluorophores, both endogenous and exogenous ones in the live system. The authors provide software based on this principle and detailed instruction to run the system. This is really attractive for the biological community as this does not require a specialized instrument and can use a hyperspectral detector with a linear array which is more commonly available to the researchers.<br><br>However, there is a major flaw that concerns me the most. The authors use only one harmonics to do phasor transformation and then linear unmixing, as stated in their mathematical arguments. This should be fine up to three components. For two components a pixel which has contribution of those components, it's phasor position is along the line joining those two components. In case of three it will be inside the triangle created by the three individual components. This isn't true for four components or higher. In four components, the position of an image phasor point can be either contributed to all four components or just three – as it will always be within a triangle. This has been shown previously by Dr. Enrico Gratton's group (PMID: 32235070). In that case another harmonic needs to be calculated to identify and explain if there are three or four components. The examples and analysis provided in this paper only involve a single harmonic – and that makes it impossible to specify the difference between image phasor points in the middle of the phasor cloud with multiple species (Fig 5F). My main concern is that use of a single harmonic assume that all of the components are present in an image pixel whose phasor coordinate is within the pentagon with the vertices occupied by the five components. It may be a very small contribution based on the fractional intensity – but it is there. This may or may not be true depending on pixel size, presence of components and basically the type of

the sample. This argument needs to be resolved as I feel this is a fundamental approach to phasor and its limitation when only one harmonic is calculated.

The editors at *Nature Methods* and *Nature Communications* would require this concern to be adequately addressed, either via justification (with evidence) or an update to the method. The editor at *Communications Biology* would also ask this to be addressed with appropriate justification.

Other comments and concerns follow below:
For all the journals, please clarify unless specified.
1. The authors provide a spectra.txt files for the unmixing. How was that obtained. How to calculate and save the data from samples having only one fluorophores?
2. I did not see any mention of collagen fluorescence when excited at 740 nm. That should be a component in the autofluorescence category.
3. What happens to FAD? That can be excited at 740 nm (PMID: 11964266).
4. About Bound NADH – does the spectra change based on the proteins it binds to?
5. What is the distance of separation of the pure components in the phasor space that allow for successful linear unmixing. I presume at some point the S/N will make it difficult if the spectra of the components are too close.
6. What about when the linear unmixing won't work? For example, a case where the individual components lie in line in the phasor space.
7. Coming back to Q1. – Prior knowledge of the components – How are they calculated? Where are the coordinates stored? Are only the center of the phasor cloud used or the whole distribution?
8. I do like the point mentioned in lines 10-20 in page 2. Spectral imaging and deconvolution is absolutely necessary.
9. Lines 30-40, page 2 – missing references about the different noise.
Please add relevant citations for all the journals.
10. The linear deconvolution of the phasor space involves fractional intensity and not the actual fraction – something that I found missing in the discussion.
11. How do 5 photons/spectra work with Poisson statistics and associated uncertainty?
12. Does the Elastin spectrum change on crosslinking in a tissue compared to the solution? **This would be nice to address but is not strictly necessary for any of the journals.**
13. Td-Tomato (PMID 19127988) and mRuby (PMID 23459413) can be excited with a 740 nm two-photon excitation. I am curious how the authors did not observe that in Figure 6.
14. What determines how much spatial denoising needs to be used? Spatial denoising indeed doesn't affect the intensity image – but it does affect the phasor mapped image – something that hasn't been discussed at all.
15. Figure 1 D-E – this is strictly not true. Once you transfer to phasor – the information remaining for the spectra is the FWHM and the peak/center – so how does the proper spectra being calculated in figure E?
16. The reduction of data from 10^7 to 10^4. How much of that is related to spectral denoising and how much is related to the transformation to phasor?
17. Page 4 line 19 – after two-components – what happens with three/four and their possible combinations?
18. One of the uses of HyU is for low light level and long term imaging.

	What happens to the deconvolution if there is bleaching? This is a minor concern. **This would be nice to address but is not strictly necessary for either of the journals.**<br>19. How to create the spectral libraries in the software provided by the authors (page 15, line 29-30)?<br>20. I do feel the references can be expanded for the phasor analysis of the multicomponent systems from other labs.
**Remarks to the Author: Impact**	The paper will influence the community - but the discrepancies need to be cleared and explained.
**Remarks to the Author: Strength of the claims**	The main concern is the linear additivity of phasor space and their implementation in this paper. Use of a single harmonic should not be enough for anything more than three components.
**Remarks to the Author: Reproducibility**	I do think the data is reproducible as the imaging is done using a commercial microscope and the authors provide an software to do so. There are details that is missing that need to be provided for the use. This includes calculation and storage of single components for the analysis.

Reviewer #2 information

**Expertise**	Fluorescence microscopy, multiplexed imaging, in vivo imaging
**Editor's comments**	Reviewer 2 thinks the work will be of great interest to those doing live imaging. However, they have a number of concerns regarding performance and presentation that should be addressed.

Reviewer #2 comments

**Section**	**Annotated Reviewer Comments**
**Remarks to the Author: Overall significance**	The manuscript of Hsiao Ju Chang et al (from the lab of Prof Cultrale) deals with dynamic (time-lapse) multiplexed imaging and offers a global-based solution for spectral unmixing of hyperspectral imaging data. Therefore, the authors improve the previously published algorithm HySP (Cultrale et al, Nat. Meth. 2017), which uses dimensionality reduction via the phasor approach (normalized discrete Fourier transformation of the hyperspectral 4D fluorescence data). They achieve this improvement by integrating in HySP a linear unmixing of the expected spectral signatures in the phase domain (HyU) - including both extrinsic signals (fluorescen proteins) and intrinsic signals (NAD(P)H, retinol, elastin, etc.). The dimensionality reduction of the phasor approach implies also a global analysis of the spectra (i.e. appreciates

	similarities of the spectra per voxel) and by that better deals with low signals. A thorough characterization of the laser, detector, read background noise and of their distribution type (Poisson, Gaussian, etc.) and implementation for denoising and additional reference-based preprocessing (SEER, Shi et al, Nat. Commun. 2020) improves not only the image quality but also the success of the hyperspectral unmixing of 8 or 9 emission (intrinsic and extrinsic) signals, at high computation speeds, as impressively demonstrated on simulated data and on time-lapse imaging data of multiple-reporter zebra fish larvae.<br><br>While being of great interest for the live imaging community, in my opinion, the manuscript needs additional experimental, algorithmic and background (citation of previous work) information to unfold the full potential, as described in detail in the following.
**Remarks to the Author: Impact**	The relevance of the question/need for simultaneous spectrally multiplexed fluorescent microscopy to allow dynamic (time-lapse) multi-color imaging is tremendous, however, certainly going far beyond the field of developmental biology and zebra fish larvae imaging. This need has been previously recognized in the frame of intravital multi-photon imaging (not hyperspectral), with impact for cancer research (Entenberg et al, 2011), immunology and neurosciences/neuroimaging, just to mention a few examples. Specifically, there have been solutions proposed and demonstrated for dynamic in vivo fluorescence imaging, including unmixing algorithms apart of the state-of-the-art linear unmixing (Rakhymzhan et al, Sci Rep 2017), in which up to 8 extrinsic and intrinsic signals are simultaneously distinguished, while dealing with low SNRs of multi-photon microscopy still remained a challenge. Including this information in the introduction is key, in order to demonstrate the potential general relevance of the present work and to awake a real interest for a broad readership.<br><br>In line with this, it is crucial to demonstrate the power of the presented algorithm for unmixing also intravital multi-photon imaging data in optically more challenging tissues and organisms, which need to deal with much lower signals and SNR values, especially due to massive scattering and wave-front distortions in mammal tissue. ***Nature Methods*** **would like to see a demonstration on a “more challenging” sample to fully explore the performance of HyU.**<br>**This is not absolutely required for *Nature Communications or Communications Biology.***<br><br>Referring to the algorithm itself and to its characterization, the evolution from hyperspectral multiplexed imaging using the phasor approach HySP (Cultrale et al, 2017, Nat Meth), enhanced by preprocessing the data to account for various experimental noise via SEER (2020, Nat Commun) and finally by applying linear unmixing in the hyperspectral phase space, bringing additional significant accuracy to the unmixing capacity of the data is currently not clear in the manuscript and needs to be elaborated in the introduction, to emphasize the novelty of the present work. **Please update the presentation for all the journals.**
**Remarks to the Author: Strength of the claims**	A. Referring to the broad applicability of the algorithm and the interest for a large community:<br><br>A.1. As previously mentioned, in order to prove the value of the approach

presented in this manuscript, multiplexed time-lapse imaging in a mammal (adult mouse or rat or human) tissue is key and experimental data on this need to be added to the manuscript. I believe, one 4D (3D + time) imaging example showing 8-9 distinct emission signals would be absolutely convincing. ***Nature Methods* would like to see a demonstration on a "more challenging" sample to fully explore the performance of HyU. *Nature Communications* do not absolutely require a more challenging demonstration but any further tissue samples would be appreciated. *Communications Biology* is happy to forego this request.**

B. Referring to the unmixing approach:
B.1. In order to judge the added value of the integration of linear unmixing and of reference extrinsic and intrinsic spectral signatures on the performance of unmixing, a thorough comparison with the previously available HySP (Cultrale et al, 2017) needs to be provided, additionally to the comparison to state-of-the-art linear unmixing algorithms already included in teh manuscript. **A comparison to HySP would be expected for *Nature Methods* and *Nature Communications*, but is not necessary for *Communications Biology*.**

B.2. A central advantage of the here presented approach is the capacity of dealing even with low signals, i.e. unmixing even low endogenous signals, such as NAD(P)H, even free and bound – having extremely similar emission spectra (one reason why their fluorescence lifetime has been used to resolve the two states). The authors show the improvement referring to number of photons per spectrum, however, in order to judge the true improvement brought by the algorithm for real imaging data (which includes background with diverse types of noise distributions), the unmixing quality needs to be related to the signal-to-noise (SNR) ratio per voxel. While mentioning SNR in the text, no values or comparison are provided in this sense – it is important to mention how the SNR as such (not only the number of photons per spectrum) impacts on the spectral resolution, i.e. how similar can be two spectra at a certain SNR to be able to still resolve them? **A clarification of how SNR affects performance, and lower bounds of spectral resolution is expected for all the journals.**

B.3. A cornerstone in acquiring better unmixing is the availability of appropriate reference spectral signatures. Whereas the current software provides the spectra necessary for the data shown in the manuscript and gives the opportunity for the users to identify signatures in their own data, the manuscript remains elusive of how the user can differentiate between a real spectral signature and different types of optical or electronical background and interferences – as well known from the use of the phasor approach in fluorescence lifetime imaging, a major challenge when dealing with experimental noisy imaging data in the frequency (phase) domain. The manuscript would benefit from including such a guide to validate the capacity for external use of the algorithm. ***Nature Methods, Nature Communications* and *Communications Biology* expect the authors to provide practical advice for using HyU with noisy data.**

B.4. Finally, fully agreeing with the authors that the number of detectors may be varied, depending on the imaged sample type and on the excitation strategy, in order to acquire an emission signal at all, an analysis of how the number of detectors (channels) impacts on the resolution between different signatures (spectra) is needed also for less than 32 detectors (4 to 6 channels being the reality in many labs due to truly low fluorescence signals

	in deep tissue, e.g. of mice or of humans). **Please clarify how the number of channels affects performance (or reference relevant literature) for *Nature Methods* and *Nature Communications*. Textually addressing this concern is enough for *Communications Biology*.**
**Remarks to the Author: Reproducibility**	The current version of the HySP platform was easy to use and the provided sample data delivered similar results as those shown in the manuscript.

Reviewer #3 information

**Expertise**	Multiplexed imaging, phasor analysis
**Editor's comments**	Reviewer 3 finds the work timely and potentially useful for biomedical imaging applications. However, they have concerns about the performance of the approach that should be addressed by revisions.

Reviewer #3 comments

Section	Annotated Reviewer Comments
**Remarks to the Author: Overall significance**	In this report, Chiang and co-workers presented the Hybrid Unmixing (HyU) method for the efficient and robust analysis of multiple fluorescent signals. The authors employ the spectral phasor method for reducing spectral data dimension and denoising noises in the imaging system. The superiority of the proposed method has been demonstrated compared to the conventional linear unmixing method by exploiting computer simulation and experimental results. This article seems to be timely the report as increasing the biomedical applications using hyperspectral imaging methods. However, I found that there are some confusing points to be addressed clearly to publish this manuscript in Nature Portfolio.<br><br>Comments:<br>1) Hyperspectral phasor compresses spectral dimension by exploiting real and imaginary parts of Fourier transformation. Moreover, there were reports that hyperspectral phasor could be applied for multiplexed fluorescence imaging. If there are any advantages of combining phasor and spectral unmixing methods, please describe them clearly in the Introduction. ***Nature Methods* and *Nature Communications* would like to see an experimental comparison of HyU with HySP (also requested by reviewer 2). Please address it textually for *Communications Biology*.**<br><br>2) If I understood correctly, numbers of photons (For instance, 5 photons per spectral in the last paragraph in Introduction) were calculated from the computer simulation. If so, this quantitative value is significantly affected by the noise levels used in the simulation. Therefore, it would be good to add these values were obtained from the simulation for clarity. **Please clarify for all the journals.**

	3) The authors addressed that the HyU method is more computationally efficient than the linear spectral unmixing method. This is true as the spectral dimension was reduced in Hyperspectral Phasors and histogram binning. However, these spectral compression and denoising also require computational power. Does the proposed method is more efficient when the entire process is considered? **Please ensure a balanced/fair comparison of overall time is included in a revision for all the journals.**<br><br>4) For spectral unmixing, it seems to use the reference signals obtained from pure fluorophores. What happens if there are unknown fluorescence signals? Can the proposed method be applied for blind spectral separation? **Please address this concern, either with simulated or experimental data for both *Nature Methods* and *Nature Communications* or discuss and cite relevant literature. Please discuss this point for Communications Biology.**<br><br>5) Following the previous question, I wonder about the effect of light scattering on the accuracy of the proposed method. In fig4, the proposed method can be applied for volumetric imaging. I wonder there are consistent fluorescence signals over the depth of tissue. Fluorescence signals occurred in deep tissue regions experience more light scattering, which might occur in spectral distortions. **As already requested, please add a more challenging demonstration on scattering tissue. We think this additional demonstration should address this concern for *Nature Methods*.**<br>**As before, *Nature Communications* do not absolutely require a more challenging demonstration, but this should be addressed in the text if experimental evidence is not possible. Address it textually for *Communications Biology*.**
**Remarks to the Author: Strength of the claims**	This work demonstrates the superiority of the proposed method using computer simulation and experimental data. The authors clearly claim that the proposed method is more efficient and robust than conventional linear spectral unmixing methods.
**Remarks to the Author: Reproducibility**	The authors provide the code and data used in the manuscript. This allows other people to reproduce these results. And the dataset used in this work is appropriate for the purpose of the study.

Open research evaluation

General information

Guidelines for Transparency and Openness Promotion (TOP) in Journal Policies and Practices ("TOP Guidelines")

The recommendations and requests in the table below are aimed at bringing your manuscript in line with common community standards as exemplified by the TOP Guidelines. While every publisher and journal will implement these guidelines differently, the recommendations below are all consistent with the policies at Nature Portfolio. In most cases, these will align with TOP Guidelines Level 2.

FAIR Principles

The goal of the recommendations in the table below related to **data or code** availability is to promote the FAIR Guiding Principles for scientific data management and stewardship (*Scientific Data* **3**: 160018, 2016). The FAIR Principles are a set of guidelines for improving 4 important aspects of digital research objects: **F**indability, **A**ccessibility, **I**nteroperability and **R**eusability.

ORCID

ORCID is a non-profit organization that provides researchers with a unique digital identifier. These identifiers can be used by editors, funding agencies, publishers, and institutions to reliably identify individuals in the same way that ISBNs and DOIs identify books and articles. Thus the risk of confusing your identity with another researcher with the same name is eliminated. **The ORCID website** provides researchers with a page where your comprehensive research activity can be stored.

Springer Nature collaborates with the ORCID organization to ensure that your research contributions (as authors and peer reviewers) are correctly attributed to you. Learn more at https://www.springernature.com/gp/researchers/orcid

Data availability
**Other data requests**
We strongly encourage the deposition of your full microscopy image data sets in the Image Data Resource: https://idr.openmicroscopy.org/about
**Code availability and citation**

To adhere to community standards and promote<br>transparency in research, any custom software or code should be made publicly available, ideally before publication so that referees can test the code and comment on it.<br><br>Please include a statement under the heading "Code Availability", indicating whether and how the custom code/software reported in your study can be accessed, including any restrictions to access. This section should also include information on the versions of any software used, if relevant, and any specific variables or parameters used to generate, test, or process the current dataset. Code availability statements should be provided as a separate section after the Data Availability section.<br><br>Upon publication, Nature Portfolio journals consider it best practice to release custom computer code in a way that allows readers to repeat the published results. Code should be deposited in a DOI-minting repository such as Zenodo, Gigantum or Code Ocean and cited in the reference list following the guidelines described in our policy pages (see link below). Authors are encouraged to manage subsequent code versions and to use a license approved by the open source initiative. Full details about how the code can be accessed and any restrictions must be described in the Code Availability statement.<br><br>See here for more information about Nature Portfolio's code availability policies:<br>https://www.nature.com/nature-portfolio/editorial-policies/reporting-standards#availability-of-computer-code<br><br>We also provide a Code and Software submission checklist that you may find useful:<br>https://www.nature.com/documents/nr-software-policy.pdf<br><br>Please note: because of advanced features used in this form, you must use Adobe Reader to open the document and complete it.
Thank you for making your custom code available via Github. Upon publication, Nature Portfolio journals consider it best practice to release custom computer code in a way that allows readers to repeat the published results. Code should be deposited in a DOI-minting repository such as Zenodo, Gigantum or Code Ocean and cited in the reference list following the guidelines described in our policy pages (see link below). Authors are encouraged to manage subsequent code versions and to use a license approved by the open source initiative.

Reporting & reproducibility

If the work involves any cancer cell lines that are listed in the database of commonly misidentified cell lines, ICLAC (http://iclac.org/databases/cross-contaminations), please provide justification for their use in the methods section. Please also state from where the lines were obtained; whether they were tested for mycoplasma contamination; and whether they were authenticated, and if so, by which method.

Statistical reporting

Data presentation: Please ensure that data presented in a plot, chart or other visual representation format shows data distribution clearly (e.g. dot plots, box-and-whisker plots). When using bar charts, please overlay the corresponding data points (as dot plots) whenever possible and always for n ≤ 10. (Please see the following editorial for the rationale behind this request and an example https://www.nature.com/articles/s41551-017-0079).

Statistics: Wherever statistics have been derived (e.g. error bars, box plots, statistical significance) the legend needs to provide and define the n number (i.e. the sample size used to derive statistics) as a precise value (not a range), using the wording "n=X biologically independent samples/animals/cells/independent experiments/n= X cells examined over Y independent experiments" etc. as applicable.

Legends requiring revision:

1. Please note that this information is missing in the legends of figures 2h; 3i and supplementary figures 4b, c; 5g.

Statistics such as error bars, significance and p values cannot be derived from n<3 and must be removed from all such cases.

We strongly discourage deriving statistics from technical replicates, unless there is a clear scientific justification for why providing this information is important. Conflating technical and biological variability, e.g., by pooling technically replicates samples across independent experiments is strongly discouraged. (For examples of expected description of statistics in figure legends, please see the following https://www.nature.com/articles/s41467-019-11636-5 or https://www.nature.com/articles/s41467-019-11510-4).

All error bars need to be defined in the legends (e.g. SD, SEM) together with a measure of centre (e.g. mean, median). For example, the legends should state something along the lines of "Data are presented as mean values +/- SEM" as appropriate.
All box plots need to be defined in the legends in terms of minima, maxima, centre, bounds of box and whiskers and percentile.

Legends requiring revision:

1. Please note that the box plots need to be defined in terms of minima, maxima, centre, bounds of box and whiskers and percentile in the legends of figures 2h; 3i and supplementary figures 4b, c; 5g.

The figure legends must indicate the statistical test used. Where appropriate, please indicate in the figure legends whether the statistical tests were one-sided or two-sided and whether adjustments were made for multiple comparisons.

For null hypothesis testing, please indicate the test statistic (e.g. F, t, r) with confidence intervals, effect sizes, degrees of freedom and P values noted.

Please provide the test results (e.g. P values) as exact values whenever possible and with confidence intervals noted.

Legends requiring revision:

1. Please indicate the statistical test used for data analysis and where appropriate, please specify whether it was one-sided or two-sided and whether adjustments were made for multiple comparisons, in the legends of figures 2h; 3i.

2. Please note that the exact p value should be provided, when possible, in the legends of figures 2h; 3i.

Reproducibility: Please state in the legends how many times each experiment was repeated independently with similar results. This is needed for all experiments, but is particularly important wherever results from representative experiments (such as micrographs) are shown. If space in the legends is limiting, this information can be included in a section titled "Statistics and Reproducibility" in the methods section.

Legends requiring revision:

Please note that this information is missing in the legends of figures 2e, i-p; 3a-f; 5a, b, e and supplementary figures 1a, b; 2a-d; 3j-m; 7a-j; 8a, b; 11a, d, g; 12a-f.

Data presentation

Micrographs: Please ensure that all micrographs include a scale bar and this scale bar is defined on the panels or in the figure legends.

Panels requiring revision:

1. Please note that the scale bar needs to be defined for supplementary figures 3j-m.

Other notes

Additional Notes

Please note that for supplementary figure 4, sequence of the
panels and their legends are incorrect. The supplementary figures ‘1d, e’ are incorrectly labelled as ‘1e, f’. Please rectify this.

Please note that the legends are not labelled as ‘6e-h’ for supplementary figure 6. Please rectify this.
